# Mitochondrial Adaptation to Diet and Swimming Activity in Gilthead Seabream: Improved Nutritional Efficiency

**DOI:** 10.3389/fphys.2021.678985

**Published:** 2021-06-18

**Authors:** Miquel Perelló-Amorós, Jaume Fernández-Borràs, Albert Sánchez-Moya, Emilio J. Vélez, Isabel García-Pérez, Joaquin Gutiérrez, Josefina Blasco

**Affiliations:** ^1^Department of Cell Biology, Physiology, and Immunology, Faculty of Biology, University of Barcelona, Barcelona, Spain; ^2^Université de Pau et des Pays de l’Adour, E2S UPPA, INRAE, UMR 1419 Nutrition Métabolisme et Aquaculture, Saint-Pée-sur-Nivelle, France

**Keywords:** high-fat, high-protein, exercise, mitochondrial dynamics, turnover, stable isotopes, *Sparus aurata*

## Abstract

Sustained exercise promotes growth in different fish species, and in gilthead seabream we have demonstrated that it improves nutrient use efficiency. This study assesses for differences in growth rate, tissue composition and energy metabolism in gilthead seabream juveniles fed two diets: high-protein (HP; 54% protein, 15% lipid) or high energy (HE; 50% protein, 20% lipid), under voluntary swimming (VS) or moderate-to-low-intensity sustained swimming (SS) for 6 weeks. HE fed fish under VS conditions showed lower body weight and higher muscle lipid content than HP fed fish, but no differences between the two groups were observed under SS conditions. Irrespective of the swimming regime, the white muscle stable isotopes profile of the HE group revealed increased nitrogen and carbon turnovers. Nitrogen fractionation increased in the HP fed fish under SS, indicating enhanced dietary protein oxidation. Hepatic gene expression markers of energy metabolism and mitochondrial biogenesis showed clear differences between the two diets under VS: a significant shift in the COX/CS ratio, modifications in UCPs, and downregulation of PGC1a in the HE-fed fish. Swimming induced mitochondrial remodeling through upregulation of fusion and fission markers, and removing almost all the differences observed under VS. In the HE-fed fish, white skeletal muscle benefited from the increased energy demand, amending the oxidative uncoupling produced under the VS condition by an excess of lipids and the pro-fission state observed in mitochondria. Contrarily, red muscle revealed more tolerant to the energy content of the HE diet, even under VS conditions, with higher expression of oxidative enzymes (COX and CS) without any sign of mitochondrial stress or mitochondrial biogenesis induction. Furthermore, this tissue had enough plasticity to shift its metabolism under higher energy demand (SS), again equalizing the differences observed between diets under VS condition. Globally, the balance between dietary nutrients affects mitochondrial regulation due to their use as energy fuels, but exercise corrects imbalances allowing practical diets with lower protein and higher lipid content without detrimental effects.

## Introduction

The growth capacity of animals is determined in a multifactorial way, involving characteristics inherent to the specific population of the reared animals as well as the physicochemical properties of the environment and the culture practices and regimes. In aquaculture, water flow is an important factor that can influence the daily energy expenditure associated with the swimming activity of fish. Thus, the study of how metabolic fuels are used to satisfy the energy demands for activity is important for the aquaculture sector to optimize feeds for optimal growth (Magnoni et al., 2013a). Exercise can stimulate growth and food conversion in various fish species (Palstra and Planas, 2013; McKenzie et al., 2020). In general, fish swimming below their maximum aerobic capacity is qualitatively similar to mammals performing aerobic exercise, showing a trend toward a more aerobic phenotype (Johnston and Moon, 1980; McClelland et al., 2006; LeMoine et al., 2010; McClelland and Scott, 2014). This positive effect of sustained moderate exercise in promoting growth also occurs in rainbow trout (Felip et al., 2012) and gilthead seabream fingerlings and juveniles (Ibarz et al., 2011; Blasco et al., 2015). This effect is regulated by the growth hormone/insulin-like growth factor (GH/IGF-I) axis (Blasco et al., 2015; Vélez et al., 2016), since there is an adjustment in the use of nutrients as the aerobic capacity of white muscle is increased and the mesenteric fat deposits are reduced. We previously demonstrated that sustained moderate swimming stimulated the use of carbohydrates, optimizing protein retention, and growth in both rainbow trout (Felip et al., 2012) and gilthead seabream (Ibarz et al., 2011; Felip et al., 2012; Martin-Perez et al., 2012). For these reasons, the exercise induced through sustained moderate swimming can be useful when formulating protein-adjusted diets, due to the more efficient use of non-protein energy. Indeed, the need to consider the formulation of fish diets in the effects of exercise has recently been pointed out (McKenzie et al., 2020), but there are not studies about it.

If growth improvement after exercise occurs through improved metabolic efficiency, this should be reflected in mitochondrial adaptation associated with the physical status of the host. The metabolism’s ability to adapt efficiently by substrate sensing, trafficking, storage, and utilization, dependent on availability and requirement, is known as metabolic flexibility (Smith et al., 2018). Mitochondria undergo constant adaptive changes in response to fluctuations in energy demand and supply, both in their absolute numbers (biogenesis) and their morphology (through fusion/fission processes), to accommodate demand for ATP (oxidative metabolism) (Liesa and Shirihai, 2013). It is known as metabolic flexibility (Smith et al., 2018). Different mitochondrial proteins participate in this process.

Peroxisome proliferator-activated receptor gamma coactivator 1-alpha, PGC1a, is a transcriptional factor that enhances mitochondrial biogenesis and oxidative function (Puigserver et al., 1998; Wu et al., 1999), being the main regulator of the cytochrome c oxidase (subunit IV of OXPHOS complex) (Scarpulla, 2011). Single bouts of exercise lead to transient increases in PGC-1a levels (Baar et al., 2002) and a high-fat diet in humans leads to decreased gene expression (Sparks et al., 2005). Uncoupling proteins (UCPs) reduce electrochemical gradients responsible for ATP generation, uncoupling oxidative phosphorylation (Divakaruni and Brand, 2011), and participate in tissue nutrient sensing and regulation of nutrient metabolism (Diano and Horvath, 2012). In response to a high-lipid diet in rats, *ucp2* upregulation and *pgc1a* downregulation have been observed (Rius-Pérez et al., 2020). Mitochondria also undergo constant fusion and fission dynamics, through changes in mitofusin (*mit1*, *mit2*) and fission (*fis1*) proteins expression. In general, fusion is stimulated where there is energy balance (demand = supply), while fission of the mitochondrial network into individual units is necessary to eliminate damaged mitochondria when cellular stress is present (Smith et al., 2018). In rats, a high fat diet induces fatty deposition in the liver and impaired mitochondrial function (Gonçalves et al., 2014, 2015), but endurance training effectively prevents these pathological processes.

Although the cellular and physiological implications of mitochondrial dynamics have been studied extensively in mammals (Westermann, 2010, 2012; Ni et al., 2015; Meyer et al., 2017; Chandhok et al., 2018), there is a scarcity of such data in fish. Mitochondrial homeostasis ensures that metabolism and physiological function persists through a global balance of mitochondrial processes (Milder and Petel, 2012). Despite extensive knowledge about this metabolic flexibility in mammals (Chen et al., 2018; Smith et al., 2018), this is not the case for fish, even though mitochondrial protein function is highly conserved. A relationship between growth, diet composition, and mitochondrial function has been observed both in channel catfish (Eya et al., 2012) and in rainbow trout (Eya et al., 2017). The influence of nutrient levels on the gene expression of respiratory chain uncoupling proteins, UCP2 and UCP3, is well documented in rainbow trout (Coulibaly et al., 2006) and gilthead seabream (Bermejo-Nogales et al., 2011, 2014), with these being the first to be modified in response to both environmental and nutritional (by caloric restriction) stressors (Bermejo-Nogales et al., 2014). Diet can modulate fusion, fission, biogenesis, and oxidation processes in yellow catfish (Song et al., 2020) and snout bream (Li et al., 2019). The ratio of cytochrome c oxidase (COX) to citrate synthase (CS) mitochondrial enzyme activity in the white muscle of exercised gilthead seabream is modulated differently depending on diet and life stage, with more protein (Blasco et al., 2015) and more carbohydrates (Martin-Perez et al., 2012) in fingerlings and juveniles, respectively (Martin-Perez et al., 2012). However, the possible regulatory role of PGC1a on oxidative metabolism is less clear than in mammals (McClelland and Scott, 2014).

Partial replacement of dietary protein with non-protein energy sources (lipids and carbohydrates) is a common practice in aquaculture, saving money on expensive ingredients and increasing nitrogen retention, and then reducing the environmental impact (by lowering nitrogen discharges). However, the efficiency of the substitution depends on endogenous factors of the fish (e.g., nutrient requirements of the species, life stage or the reproductive phase). Moreover, high-energy diets for aquaculture may reduce the period until the market size is reached for some species, and decrease feed costs by reducing the amount of money spent on proteins (Leaver et al., 2008). In fish, however, undue increases in lipid content have been associated with high fat deposition (Caballero et al., 1999; Company et al., 1999) and increased oxidative stress (Sánchez-Nuño et al., 2018). An optimal diet should provide the necessary nutrients in appropriate proportions for maintenance, tissue repair and growth, without excessive deposits of fuel reserves. Stable isotope analysis of fish nutrition has been revealed to be useful in the evaluation of reserve turnover (Martínez Del Rio et al., 2009) because enzymes involved in catabolic processes, such as decarboxylation and deamination show a preference for light isotopes (Gannes and Marti, 1998). This causes tissues to become enriched with heavier isotopes (e.g., ^13^C and ^15^N). This factor of discrimination is known as fractionation. We have evaluated the use of stable isotopes as an indicator of feeding balance when assessing the optimal nutritional conditions for growing fish (Beltrán et al., 2009; Martín-Pérez et al., 2011; Martin-Perez et al., 2012; Martin-Pérez et al., 2013). Our studies have demonstrated that nitrogen dietary fractionation (Δδ^15^N) is a good marker of protein balance because it reflects protein turnover and retention efficiency (Martínez Del Rio et al., 2009; Martín-Pérez et al., 2011). In gilthead seabream, we observed an inverse relationship between dietary protein content and muscle nitrogen fractionation, and that this fractionation was also inversely related to the specific growth rate (SGR) (Martin-Pérez et al., 2013). This reflects a direct relationship between diet protein content and SGR.

The practice of using lipid supplementation to decrease dietary protein levels in order to reduce production costs is not common during the early life-stages because it may cause imbalances that negatively affect these periods of rapid growth and compromise the efficiency of the culture and the quality of the product. Understanding how changes in diet composition related to swimming activity affect mitochondria can help to clarify the molecular mechanisms that underpin growth performance and body composition. Therefore, the aim of the present study was to analyze the beneficial effects of sustained swimming in gilthead seabream fingerlings that were fed commercial diets with different proportions of macronutrients (substitution of proteins with lipids), especially their effects on the mitochondrial homeostasis of the main tissues. We measured growth parameters, body indices and the principal components of the main tissues (liver, and white and red muscle). Since the balance and availability of nutrients may affect cell metabolism, we analyzed the gene and protein expression of mitochondrial proteins and the ^15^N and ^13^C isotope relationships in the liver and both types of skeletal muscle. Two groups of fish maintained under similar conditions that performed voluntary swimming were also analyzed for the same variables, acting as the reference values for the diets.

## Materials and Methods

### Experimental Design

Nine hundred eighty gilthead seabream fingerlings (mean body weight, 4.1 ± 0.1 g) were obtained from a commercial hatchery (Piscimar, Burriana, Spain) and reared in the facilities of the Faculty of Biology (University of Barcelona) at 23 ± 1°C under a 15L:9D photoperiod. After 1 week of acclimation, the fish were anesthetized with MS-222 and their individual weight and length measured. The fish were distributed between eight 200-L (*n* = 80 for each tank) and two 400-L (*n* = 160 for each tank) tanks at the same biomass density (1.5 kg/m^3^). A representative number of fish (40 undertaking voluntary swimming (VS) and 120 performing sustained swimming (SS) for each diet group) were fitted with a passive integrated transponder (PIT, size:1.25 mm × 7 mm) tag (Trovan Electronic Identification Systems, Madrid, Spain) near the dorsal fin to enable their identification and individual monitoring of the SGR. The 400-L tanks were kept in rearing conditions, where the fish swam spontaneously (voluntary swimming group, VS). In the 200-L tanks, a circular laminar flow was created, placing a plastic column at the center of each tank and using a plastic flute to direct the water exit in a tangential direction toward the wall of the tank. The flow of each tank was regulated to achieve an initial speed of 2.5 body lengths (BL)/second to force the fish to perform sustained moderate swimming (sustained swimming group, SS). The flow speed of these tanks was adjusted during the experiment to maintain the speed as the fish body length increased. The fish food was obtained from Skretting España S.A. (Burgos, Spain), and two different diets were chosen based on their composition (Table 1). One was the high protein diet (HP diet, 54P/15L), while the other was the high energy diet (HE diet, 50P/20L). Each diet was used to feed half (one 400-L and four 200-L) of the tanks. The daily ration was set as 5% of the total biomass for each tank divided into three meals. The pellet size was increased 3 weeks after the beginning of the trial.

**TABLE 1:** Chemical composition of the diets.

	HP DIET	HE DIET
Digestible energy (MJ/kg)	18	19.9
Protein (% DM)	54	50
Lipids (% DM)	15	20
DHA (% DM)	15	20
EPA (% DM)	1	1.4
ARA (% DM)	2.5	3
DHA/EPA/ARA	5/12.5/1	3.5/7.5/1
Cellulose (% DM)	1.6	1.7
Ash (% DM)	10.5	6.4
Total P (% DM)	1.4	1.2
Estimated nitrogen-free extract	20.5	23.5

*DM, dry matter; DHA, docosahexaenoic acid; EPA, eicosapentaenoic acid; ARA, arachidonic acid; P, elemental phosphorus.*

The biometric parameters (weight and standard length) of all the fish were measured at the end of the experiment (6 weeks) and used to calculate the Condition Factor (CF) [CF = W/L^3^], where W is the body weight in grams and L is the body length in cm. At this point and after 12 h of fasting, the PIT-tagged fish from each tank were sacrificed by sectioning the spinal cord and eviscerated. The weights of the mesenteric fat and liver were obtained, and the mesenteric fat index (MFI) and hepatosomatic index (HSI) were calculated. Specific growth rate was calculated for each PIT-tagged fish [SGR = 100 × ((ln (Wf) - ln (Wi))/T)], where Wf and Wi are the final and initial body weight of the fish in grams, respectively, and T is the time of experiment in days. Samples of liver and red and white skeletal muscle were taken and stored in liquid nitrogen until further analysis.

All animal handling procedures were conducted following the norms and procedures established by the European Union Council (86/609/EU) and the Spanish and Catalan Governments, with approval obtained from the Ethics and Animal Care Committee of the University of Barcelona (permit number DAAM 7644).

### Muscle Proximate Composition and Isotopic Composition Analysis (δ^15^N and δ^13^C)

Samples of white muscle were ground in liquid N_2_ using a pestle and mortar to obtain a fine powder. Aliquots of each sample were taken for use in isotopic analyses and to assess the lipid, protein, glycogen, and water contents. Water content was determined gravimetrically after drying the samples at 95°C for 24 h. Lipids were extracted as described by Bligh and Dyer (1959). The lipid extracts were dried under an N_2_ atmosphere and total lipids determined gravimetrically. Proteins were purified from defatted tissue samples via precipitation with 10% (v/v) trifluoroacetic acid. The extracts were dried using a vacuum system (Speed Vac Plus AR, Savant Speed Vac Systems, South San Francisco, CA, United States) and the protein content was calculated from the total N content obtained by elemental analysis (Elemental Analyzer Flash 1112, Thermo Finnigan, Bremen, Germany), assuming 1 g of N for every 6.25 g of protein. Glycogen was extracted and purified from the tissues following alkaline hydrolysis by boiling with 30% KOH and an alcoholic precipitation, as described by Good and coworkers (Good et al., 1933). The glycogen content was then assessed using the anthrone-based colorimetric method described by Fraga (1956).

Samples of diet and white muscle were lyophilized and ground into a homogenous powder for isotopic analysis. Aliquots of the diet and their purified fractions (lipid and protein) and of white muscle and their purified tissue fractions (glycogen, lipid, and protein), which ranged from 0.3 to 0.6 mg, were weighed in small tin capsules. Samples were analyzed to determine the carbon and nitrogen isotope composition using a Mat Delta C isotope ratio mass spectrometer (Finnigan MAT, Bremen, Germany) coupled to a Flash 1112 Elemental Analyzer. Isotope ratios (15N/14N, 13C/12C) determined by isotope ratio mass spectrometry are expressed in delta (δ) units (parts per thousand, ‰), as follows:

δ=[(Rsa/Rst)-1]×1000

where, Rsa is the 15N/14N or 13C/12C ratio of the samples and Rst is the 15N/14N or 13C/12C ratio of the international standards (Vienna Pee Dee Belemnite for C and air for N). The same reference material analyzed during the experimental period was measured with ± 0.2‰ precision. Nitrogen isotopic fractionation values (Δδ^15^N) were calculated as the difference between the δ value of the tissue and the corresponding δ value of the diet.

### RNA Extraction, cDNA Synthesis, and q-PCR

To perform the gene expression analysis, tissue homogenization was performed using 100 mg of white muscle. Tissue samples were homogenized in 1 ml of TRI Reagent^®^ (Applied Biosystems, Alcobendas, Spain), using the Precellys^®^ Evolution Homogenizer cooled with Cryolys^®^ (Bertin-Corp, Montigny-le-Bretonneux, France) at 4–8°C. After homogenization, RNA extraction was performed following the protocol of the TRI Reagent^®^ manufacturer. The RNA concentration of each sample was measured using Nanodrop2200 (Thermo Fisher Scientific, Alcobendas, Spain). The RNA integrity of each sample was checked using 1% agarose gel electrophoresis with 3% SYBR^®^ Safe DNA Gel Stainer (Bio-Rad, El Prat de Llobregat, Spain). Then, one μg of RNA was treated with DNase I (Life Technologies, Alcobendas, Spain) and retrotranscribed with the Transcriptor First Strand cDNA Synthesis Kit (Roche, Sant Cugat del Vallès, Spain).

Gene expression analysis was performed by quantitative polymerase chain reaction (q-PCR) with the cDNA samples according to the requirements of the MIQE guidelines (Bustin et al., 2009), using the iTAQ Universal SYBR^®^ Green Supermix (Bio-Rad, El Prat de Llobregat, Spain) in Hard-Shell^®^ 384-well PCR plates (Bio-Rad, El Prat de Llobregat, Spain) and the CFX384TM Real-Time System (Bio-Rad, El Prat de Llobregat, Spain). The q-PCR program was: 3 min at 95°C, 39 × (30 s at 95°C, 30 s at primer melting temperature and fluorescence detection), 5 s at 55°C followed by a melting curve ranging from 55 to 95°C with an increase of 0.5°C every 30 s.

The primer sequences and GenBank accession numbers are shown in the Table 2. The reference genes elongation factor 1 alpha (*ef1*α), ribosomal protein S18 (*rps18*), ribosomal protein L27 (*rpl27*), and mitochondrial import receptor subunit TOM20 (*tom20*) were analyzed and the combination of the most stable ones was used to calculate the relative expression of the genes of interest following the Pfaffl method (Pfaffl, 2001). The stability of the reference genes (assessed with the geNorm algorithm) and the relative expression to the geometric mean of the reference genes were calculated with the Bio-Rad CFX Manager^TM^ 3.1 software.

**TABLE 2:** Primers used for real-time quantitative PCR.

Gene	Sequences 5′-3′	Ta (°C)	Accession number
*cox4a*	F: ACC CTG AGT CCA GAG CAG AAG TCC R: AGC CAG TGA AGC CGA TGA GAA AGA AC	60	JQ308835
*cs*	F: TCC AGG AGG TGA CGA GCC R: GTG ACC AGC AGC CAG AAG AG	60	JX975229
*pgc1a*	F: CGT GGG ACA GGT GTA ACC AGG ACT C R: ACC AAC CAA GGC AGC ACA CTC TAA TTC T	60	JX975264
*ucp1*	F: GCA CAC TAC CCA ACA TCA CAA G R: CGC CGA ACG CAG AAA CAA AG	60	FJ710211
*ucp2*	F: CGG CGG CGT CCT CAG TTG R: AAG CAA GTG GTC CCT CTT TGG TCA T	60	JQ859959
*ucp3*	F: AGG TGC GAC TGG CTG ACG R: TTC GGC ATA CAA CCT CTC CAA AG	60	EU555336
*mit1*	F: CAT CGT TGG AGG AGT GGT GTA R: CCG TAC AGT GAG GCT GAG AG	60	JX975250
*mit2*	F: GGG ATG CCT CAG CCT CAG AAC CT R: CTG CCT GCG GAC CTC TTC CAT GTA TT	60	JX975251
*fis1*	F: TCT CAG GAA CGA GCC AGG GAA CA R: CCT TGT CGA TGA GTT TCT CCA GGT CCA G	60	JX975249
*miffb*	F: CGC AGC AGC ATT CCC TTC R: CTC GTA CTG GAT TCG GTT CAT CT	60	JX975252
*ef1a*	F: CTTCAACGCTCAGGTCATCAT R: GCACAGCGAAACGACCAAGGGGA	60	AF184170
*rps18*	F: GGGTGTTGGCAGACGTTAC R: CTTCTGCCTGTTGAGGAACCA	60	AM490061.1
*tom20*	F: TGT TCA TCG GGT ACT GCA TC R: TTC TGC TTT CTC CTC CGT TC	60	FM146454.1
*rpl27*	F: AAGAGGAACACAACTCACTGCCCCAC R: CTTCTGCCTGTTGAGGAACCA	68	AY188520

*Ta, annealing temperature in the qPCR.*

### Protein Extraction and Western Blot Analysis

Protein was extracted from 100 mg of white skeletal muscle in 1 mL of RIPA buffer supplemented with phosphatase (PMSF and NA_3_VO_4_) and protease inhibitors (P8340, Santa Cruz) using the Precellys^®^ Evolution homogenizer coupled to a Cryolys cooling system (Bertin Technologies, Montigny-le-Bretonneux, France).

Soluble protein concentration was determined by the Bradford method, using bovine serum albumin (BSA) (Sigma Aldrich, Tres Cantos, Spain) for the standard curve. 20 μg of the soluble protein fraction were added to a loading buffer (containing SDS and β-mercaptoethanol), heated at 95°C for 5 min and run in a 12% polyacrylamide gel. The proteins were then transferred overnight to Immobilon^®^-FL PVDF 0.2-μm transfer membranes (Merck Millipore Ltd., Tullagreen, Cork, Ireland) that had been previously activated in methanol. Total transferred protein was determined by a 5-min incubation with REVERT^TM^ Total Protein Stain (LI-COR, Lincoln, Nebraska, United States). The signal was read at 700 nm using the Odyssey Fc Imaging System (LI-COR). After total protein quantification, the membranes were blocked with Odyssey Blocking Buffer (diluted 1:1 in TBS) (LI-COR) for 1 h at room temperature before being incubated overnight at 4°C and in agitation with the corresponding primary antibody diluted in blocking buffer + 0.05% Tween20. The primary antibodies used were purchased from ABCAM (Cambridge, United Kingdom), as follows: rabbit polyclonal anti-COX IV antibody (ab16056; 1:1000), rabbit polyclonal anti-CS antibody (ab96600; 1:2000), mouse monoclonal anti-mitofusin 1 + mitofusin 2 antibody [3C9] (ab57602; 1/1000) and rabbit polyclonal anti-UCP3 antibody (ab180643; 1/500 for WM and 1/1000 for RM). The cross-reactivity of these antibodies with gilthead seabream was confirmed by the molecular weight of the bands. In the case of COX4a and CS, the blots were performed from the same membranes which were split prior to the primary antibody incubation. After washing with TBS-T, the membranes were incubated with the corresponding secondary antibodies diluted in blocking buffer + 0.05% Tween20 at 1:10000 dilution: IRDye^®^ 800CW Goat anti-Rabbit (925-32211), Li-Cor, Lincoln, Nebraska, United States) and IRDye^®^ 800CW Goat anti-Mouse (925-32210, Li-Cor, Lincoln, Nebraska, United States). After incubation, the membranes were washed with TBS-T and the fluorescence of the immunoreactive bands was measured at 800 nm using the Odyssey Fc Imaging System (LI-COR). Stripping was performed using a commercial stripping buffer (NewBlot PVDF 5X Stripping Buffer) (LI-COR).

### Statistics

Data for all parameters are presented as means ± standard error of the mean (SEM). Data normality and homoscedasticity through groups were checked with the Shapiro–Wilk test followed by Levene’s test. Data were analyzed by a two-way analysis of variance (ANOVA) with diet (HP, HE) and swimming activity (VS, SS) set as independent factors, and their interaction. Pairwise comparisons were analyzed by unpaired student *t*-tests. Differences among groups were considered significant at *p* < 0.05. Data were analyzed using IBM SPSS version 25 (IBM Corp., Armonk, NY, United States).

## Results

### Somatic Parameters and Muscle Composition

Table 3 shows the results for somatic parameters. Diet type affected final body weight, CF, and SGR, with an interaction being present for the two first parameters. The HP diet group presented a significantly higher body weight and lower CF than the HE diet group. Swimming activity significantly increased body weight in the HE diet group, with observed differences between the two groups disappearing. The CF also decreased with the HE diet, but differences were maintained. The final body weight of the PIT-tagged fish was comparable to the body weight based on the total biomass of each group (VS: HP = 17.90 ± 0.31, HE = 15.95 ± 0.38, *p* < 0.001; SS: HP = 16.12 ± 0.48, HE = 15.94 ± 0.48, ns). Diet affected the HSI, with this being significantly higher in HE group. The MFI was also significantly higher in the HE group under the SS condition, because significant interaction was observed.

**TABLE 3:** Somatic growth parameters of gilthead seabream fed the HP or HE diet under conditions of voluntary swimming (VS) or sustained swimming (SS).

	VS		SS		
	HP DIET	HE DIET	HP DIET	HE DIET	
BW	16.42 ± 0.25	15.01 ± 0.21***	16.12 ± 0.20	15.99 ± 0.17^†††^	D: < 0.001 A: NS D × A: 0.003
CF	1.46 ± 0.01	1.54 ± 0.01***	1.54 ± 0.01^†††^	1.52 ± 0.01**^†^	D: < 0.001 A: < 0.001 D × A: < 0.001
HSI	1.42 ± 0.04	1.93 ± 0.05***	1.55 ± 0.03^†††^	1.89 ± 0.06***	D: < 0.001 A: NS D × A: NS
MFI	1.20 ± 0.10	1.07 ± 0.08	1.01 ± 0.09	1.29 ± 0.10*	D: NS A: NS D × A: 0.024
SGR	3.24 ± 0.06	3.19 ± 0.04	3.14 ± 0.03	3.05 ± 0.03*^†^	D: 0.010 A: NS D × A: NS

Data are presented as the mean ± SEM. Body weight (BW) and Condition factor: *n* = 154 for VS and *n* = 315 for SS conditions for each diet group (all fish). Hepatosomatic index (HSI), mesenteric fat index (MFI), and specific growth rate (SGR): *n* = 40 for VS and *n* = 120 for SS conditions for each diet group (PIT-tagged fish). The *p*-values of the two-way ANOVA analysis are displayed in the right column. D, diet; A, Activity; D × A, Interaction; n.s., *p*-value > 0.05. Pairwise comparisons were assessed by unpaired Student’s *t*-test. *p < 0.05, **p < 0.01, and ***p < 0.001 between diet groups (HP and HE). ^†^*p* < 0.05 and ^†††^*p* < 0.001 between swimming activity groups (VS and SS).

Regarding muscle composition (Table 4), diet affected the lipid content of white muscle (significantly higher in the HE diet group) and swimming activity significantly decreased muscle glycogen content, irrespective of diet. Protein content was not affected by any factor. The isotopic composition (δ^13^C and δ^15^N) of muscle and its reserves was significantly different between the HP and HE diet groups, regardless of the swimming condition (Table 4). The δ^15^N and δ^13^C values of bulk muscle, and its components (δ^15^N-protein, δ^13^C-glycogen, and δ^13^C-lipid), were significantly lower in the HE diet group compared with the HP diet group (δ^15^N: HP diet, 8.07 ± 0.06; HE diet, 6.97 ± 0.09). Nitrogen fractionation (Δδ ^15^N) was significantly higher in the HE diet group, indicating a higher protein turnover in fish fed the HE diet, but the difference between the two diet groups decreased under SS conditions (Δδ^15^N HP – Δ^15^N HE: 3.42–3.88 = −0.46 in VS; 3.52–3.86 = −0.34 in SS) because of greater fractionation in the HP diet group.

**TABLE 4:** Proximal composition and stable isotopes analysis in skeletal white muscle.

	VS		SS		
	HP DIET	HE DIET	HP DIET	HE DIET	
% moisture	77.41 ± 0.13	77.25 ± 0.20	77.31 ± 0.22	77.17 ± 0.16	D: NS A: NS D × A: NS
% glycogen	0.37 ± 0.02	0.35 ± 0.03	0.27 ± 0.02^†^	0.28 ± 0.02^†^	D: NS A: < 0.001 D × A: NS
% lipid	1.45 ± 0.02	1.55 ± 0.02***	1.47 ± 0.03	1.54 ± 0.01	D: < 0.001 A: NS D × A: NS
% protein	21.22 ± 0.24	20.95 ± 0.24	21.05 ± 0.025	21.02 ± 0.37	D: NS A: NS D × A: NS
δ^15^N DM^1^	11.52 ± 0.07	10.85 ± 0.08***	11.62 ± 0.05	10.84 ± 0.07***	D: < 0.001 A: NS D × A: NS
δ^13^C DM^1^	−19.33 ± 0.03	−20.49 ± 0.05***	−19.43 ± 0.04	−20.49 ± 0.03***	D: < 0.001 A: NS D × A: NS
δ^13^C glycogen	−20.74 ± 0.12	−21.89 ± 0.12***	−20.92 ± 0.12	−21.73 ± 0.13***	D: < 0.001 A: NS D × A: NS
δ^13^C lipid	−24.45 ± 0.18	−25.08 ± 0.08**	−24.20 ± 0.06	−25.09 ± 0.05***	D: < 0.001 A: NS D × A: NS
δ^15^N protein	12.56 ± 0.06	11.81 ± 0.10**	12.74 ± 0.02^†^	11.92 ± 0.07***	D: < 0.001 A: NS D × A: NS
Δδ^15^N DM^1^	3.42 ± 0.07	3.88 ± 0.08***	3.52 ± 0.06	3.86 ± 0.07***	D: < 0.001 A: NS D × A: NS

Data are presented as the mean ± SEM (*n* = 12). The *p*-values of the two-way ANOVA analysis are displayed in the right column. D, diet; A, activity; D×A, interaction; n.s., *p*-value > 0.05. Pairwise comparisons were assessed by unpaired Student’s *t*-test. **p < 0.01 and ***p < 0.001 between diet groups; ^†^*p* < 0.05 between physical activity groups. ^1^DM, dry matter. Δδ^15^N, nitrogen fractionation, was calculated based on the δ^15^N DM measured for both diets, which were 8.07 ± 0.6 and 6.97 ± 0.09 for the HP and HE diets, respectively.

### Gene Expression

#### Liver

**FIGURE 1:**
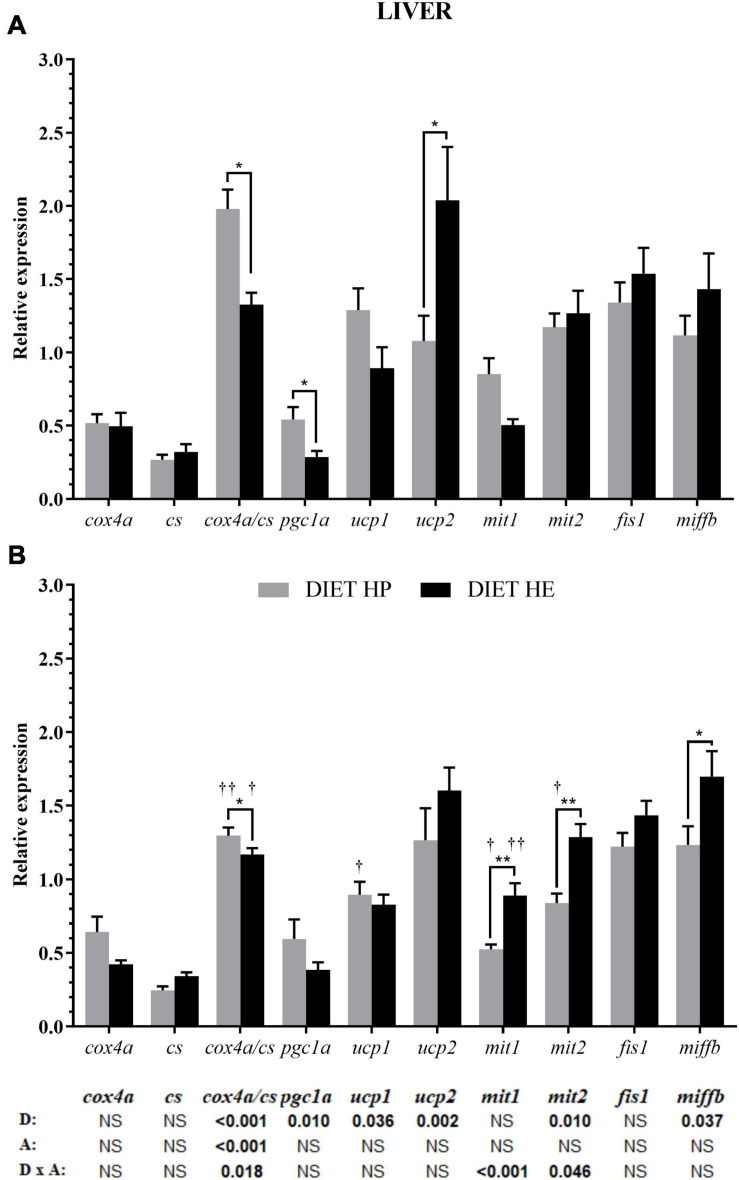
Relative gene expression of the enzymes in energy metabolism, markers of mitochondrial biogenesis and markers of mitochondrial dynamics in the liver of gilthead seabream fed the HP or HE diet under conditions of voluntary swimming **(A)** or sustained swimming **(B)**. Data are shown as the mean ± SEM (*n* = 12 for VS and *n* = 16 for SS conditions). The factorial statistical analysis was assessed by two-way ANOVA and the *p*-values of the diet (D), swimming activity (A) and the interaction (D × A) are displayed at the bottom of the figure. Pairwise comparisons were assessed by unpaired Student’s *t*-test. *p < 0.05 and **p < 0.01 between diet groups (HP AND HE); ^†^*p* < 0.05 and ^††^*p* < 0.01 between swimming activity groups (VS and SS).

Diet modified the expression of genes for mitochondrial proteins related to energy metabolism (*ucps*, *pgc1a, and cox4a/cs* ratio) and mitochondrial fusion/fission (*mit2* and *miffb*) (bottom of Figure 1). Despite the lack of significant differences in *cox* and *cs* gene expression by diet or activity, minor changes in these genes caused significant effects over their ratio (*cox4a/cs*) by both factors as well as their interaction. Interestingly, the uncoupling proteins *ucp1* and *ucp2* displayed inverse expression patterns. The gene expressions of *pgc1a* and *ucp1* were higher in fish fed the HP diet, while *ucp2* expression was higher in fish fed the HE diet (Figure 1A). Lower *mit2* and *mffb* gene expressions were found in the HP diet group, but under SS conditions, a significant interaction was found for *mit1* and *mit2* expressions, resulting in higher expression in the HE diet group (Figure 1B).

#### White and Red Skeletal Muscle

**FIGURE 2:**
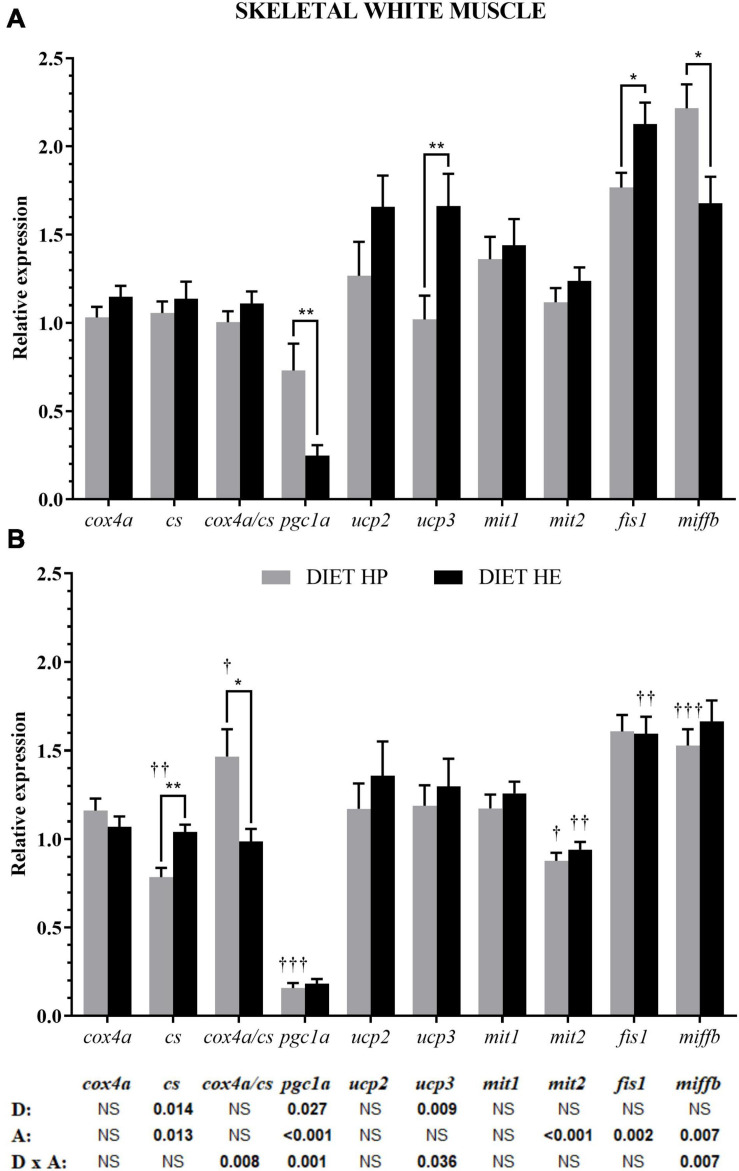
Relative gene expression of the enzymes in energy metabolism, markers of mitochondrial biogenesis, and markers of mitochondrial dynamics in the white skeletal muscle of gilthead seabream fed the HP or HE diet under conditions of voluntary swimming **(A)** or sustained swimming **(B)**. Data are shown as the mean ± SEM (*n* = 12 for VS and *n* = 16 for SS conditions). The factorial statistical analysis was assessed by two-way ANOVA and the *p*-values of the diet (D), swimming activity (A) and the interaction (D × A) are displayed at the bottom of the figure. Pairwise comparisons were assessed by unpaired Student’s *t*-test. ^∗^*p* < 0.05 and ^∗∗^*p* < 0.01 between diet groups (HP and HE); ^†^*p* < 0.05, ^††^*p* < 0.01, and ^†††^*p* < 0.001 between swimming activity groups (VS and SS).

In white muscle, both diet and swimming activity induced changes in the expression of genes related to energy metabolism, but only activity modified the fusion/fission proteins (bottom of Figure 2). Diet modified the expressions of *cs, pgc1a*, and *ucp3*. Under VS conditions, *pgc1a* expression in fish fed the HP diet was significantly higher than in those fed the HE diet, whereas the reverse pattern was true of *ucp3* expression (Figure 2A). The expressions of *cs* and *pgc1a*, as well as three proteins related to fusion (*mit1*) and fission (*fis1* and *miffb*), were also modified by activity (Figure 2B). Interaction was observed for *cox/cs* ratio, *pgc1a*, *ucp3*, and *miffb.* Activity significantly decreased the expressions of *cs* and *pgc1a* in the HP group, explaining the observed interaction for these and *ucp3* in the HE group. Activity significantly decreased *mit2* expression in both diet groups, *fis1* expression in the HE group, and *miffb* expression in the HP group; this latter gene showed interaction.

**FIGURE 3:**
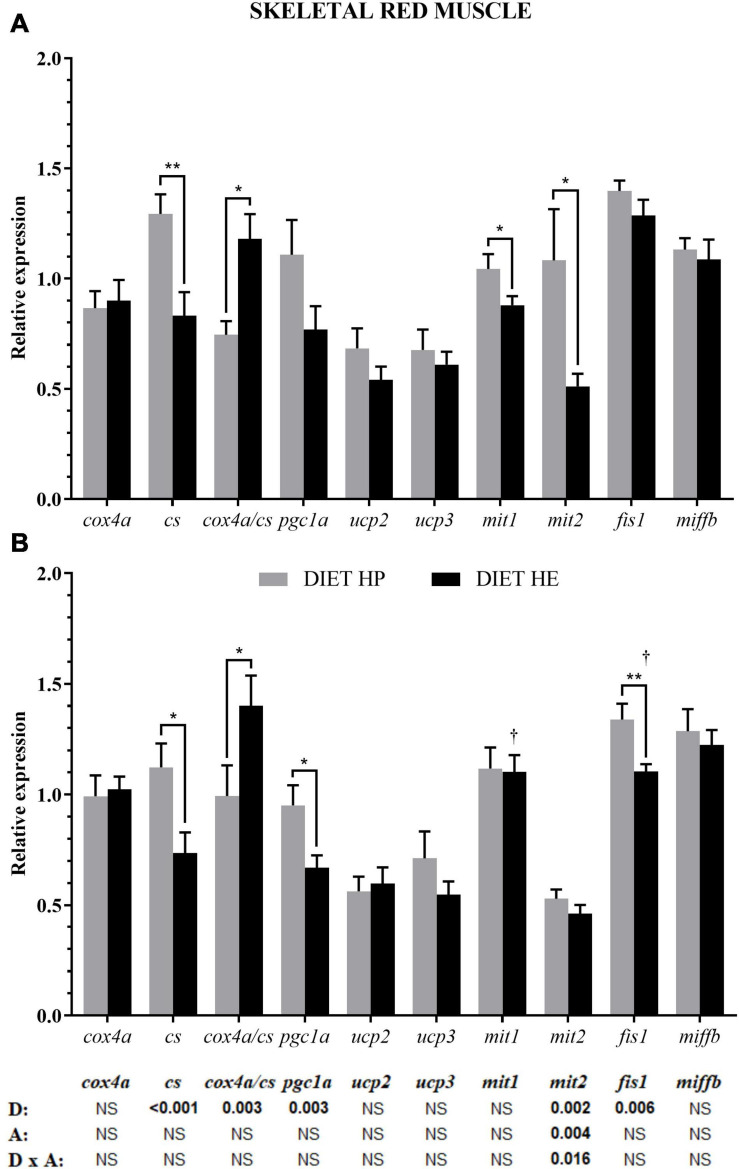
Relative gene expression of the enzymes in energy metabolism, markers of mitochondrial biogenesis and markers of mitochondrial dynamics in the red skeletal muscle of gilthead seabream fed the HP or HE diet under conditions of voluntary swimming **(A)** or sustained swimming **(B)**. Data are shown as the mean ± SEM (*n* = 12 for VS and *n* = 16 for SS conditions). The factorial statistical analysis was assessed by two-way ANOVA and the *p*-values of the diet (D), swimming activity (A) and the interaction (D × A) are displayed at the bottom of the figure. Pairwise comparisons were assessed by unpaired Student’s *t*-test. ^∗^*p* < 0.05 and ^∗∗^*p* < 0.01 between diet groups (HP and HE); ^†^*p* < 0.05 between swimming activity groups (VS and SS).

In red muscle, diet modified *cs* and *pgc1a* expression related to energy metabolism, *mit2* expression related to fusion, and *fis1* expression related to fission. Activity significantly modified the expressions of *mit2* (bottom of Figure 3) and *fis1* (*p* < 0.052). The HP group had significantly higher *pgc1a* and *cs* expressions, and a lower *cox4a/cs* ratio, compared with the HE group (Figures 3A,B). Notably, *mit2* expression was significantly higher in the HP group, but the difference was lost with activity (Figure 3B) due to interaction. Because of the swimming activity, a significant reduction in *fis1* expression was found in the HE group under the VS condition, being significantly lower than in the HP group (Figure 3B).

### Protein Expression

#### White Skeletal Muscle

**FIGURE 4:**
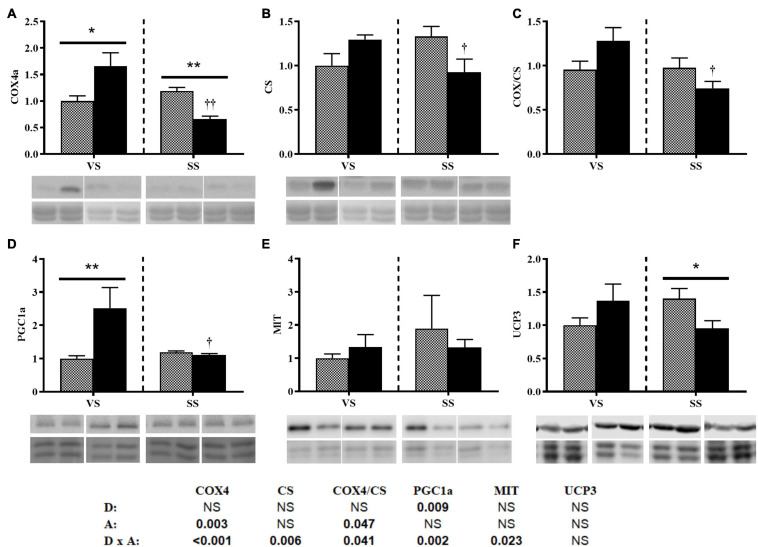
Representative western blot and densitometric analysis of the protein levels of COX4 **(A)**, CS **(B)**, the COX/CS ratio **(C)**, PGC1a **(D)**, MIT1/2 **(E)**, and UCP3 **(F)** in the white skeletal muscle of gilthead seabream fed the HP or HE diet under conditions of **(A)** voluntary swimming (VS) or **(B)** sustained swimming (SS). Bands were normalized to their Revert^TM^ total protein staining (the corresponding well is shown). To eliminate intermembrane variability, the relative intensity of each specific band was normalized by the geometric mean of the intensity of that of the VS HP diet group of the corresponding membrane. For COX4a and CS, the blots were performed from the same membranes which were split before the primary antibody incubation. Data are shown as the mean ± SEM (*n* = 6). The factorial statistical analysis was assessed by two-way ANOVA and the *p*-values of the diet (D), swimming activity (A) and the interaction (D × A) are displayed at the bottom of the figure. Pairwise comparisons were assessed by unpaired Student’s *t*-test. ^∗^*p* < 0.05 and ^∗∗^*p* < 0.01 between diet groups (HP and HE); ^†^*p* < 0.05 and ^††^*p* < 0.01 between swimming activity groups (VS and SS).

Diet only modified the protein expression of PGC1a, and there was an interaction with activity (bottom of the Figure 4). Under the VS condition, the HE group presented significantly higher levels of PGC1a, whereas under the SS condition, PGC1a levels were significantly decreased (i.e., comparable between the two diet groups) (Figure 4D). Activity affected COX levels, and consequently, the COX/CS ratio changed significantly (Figures 4A–C). Both COX and CS levels, and their ratio, showed interactions (bottom of Figure 4). Under the VS condition, COX4 protein levels were significantly higher in the HE group. However, COX and CS levels decreased significantly in the HE group under SS conditions compared with VS conditions. Interaction was observed in UCP3 protein levels (Figure 4F).

#### Red Skeletal Muscle

**FIGURE 5:**
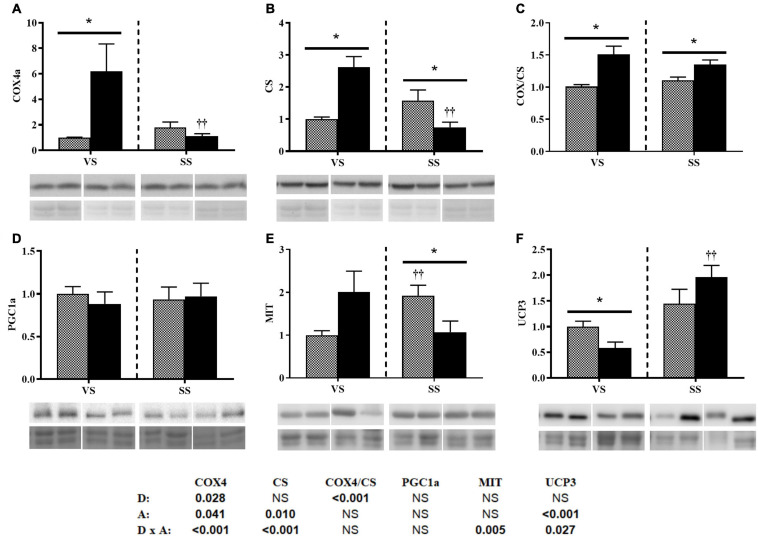
Representative western blot and densitometric analysis of the protein levels of COX4 **(A)**, CS **(B)**, the COX/CS ratio **(C)**, PGC1a **(D)**, MIT1/2 **(E)**, and UCP3 **(F)** in the red skeletal muscle of gilthead seabream fed the HP or HE diet under conditions of **(A)** voluntary swimming (VS) or **(B)** sustained swimming (SS). Bands were normalized to their Revert^TM^ total protein staining (the corresponding well is shown). To eliminate intermembrane variability, the relative intensity of each specific band was normalized by the geometric mean of the intensity of that of the VS HP diet group of the corresponding membrane. For COX4a and CS, the blots were performed from the same membranes which were split before the primary antibody incubation. Data are shown as the mean ± SEM (*n* = 6). The factorial statistical analysis was assessed by two-way ANOVA and the *p*-values of the diet (D), swimming activity (A) and the interaction (D × A) are displayed at the bottom of the figure. Pairwise comparisons were assessed by unpaired Student’s *t*-test. ^∗^*p* < 0.05 between diet groups (HP and HE); ^††^*p* < 0.01 between swimming activity groups (VS and SS).

In red muscle, diet only affected COX4 levels, and this resulted in changes to the COX/CS ratio (bottom of Figure 5). Activity altered the levels of COX, CS (Figures 5A–C), and UCP3 (Figure 5F), related to energy metabolism. Diet and activity interacted for the three proteins (bottom of Figure 5). Under VS conditions, COX4 and CS levels, as well as the COX/CS ratio, were significantly higher and UCP3 levels were significantly lower in the HE group; by contrast, SS conditions significantly decreased these enzyme levels and increased UCP3 levels in this group. Activity led to increased MIT levels in the HP group, with these being significantly higher than in the HE group; an interaction was observed between the two factors (Figure 5E). PGC1a synthesis was not affected by diet or activity (Figure 5D).

## Discussion

### Somatic Parameters and Muscle Composition

It has been shown for some fish species that sustained moderate exercise promotes growth and improves food conversion rates in several teleost fish species (Davison, 1997; Palstra and Planas, 2013; Blasco et al., 2015). However, the potential growth enhancement induced by this type of exercise is strictly related to the natural physical activity pattern of each species, showing effects that are more marked in pelagic fish species (Palstra and Planas, 2013). Recently, it has been postulated that the beneficial effects of exercise on growth are achieved when the swimming speed is close to the optimal swimming speed of each species (U*opt*) (McKenzie et al., 2020), with the feed intake covering all the energy costs of swimming. It has been established that the U*opt* of gilthead seabream juveniles weighing 25–45 g is 4.5 BL⋅s^–1^ (Palstra et al., 2020). As the fish in the present study were 2–4 g, their U*opt* should be around 5 BL⋅s^–1^ (0.35–0.4 m⋅s^–1^). In the present study, sustained moderate-to-low-intensity exercise at a speed near 50% of the U*opt* was used (as this speed is considered more practical for fish farming) to determine if the different proportions of protein and lipid in commercial diets met the increased metabolic demands of swimming activity without compromising growth.

The different composition of the diets used (HP and HE diets) was reflected in the growth and body composition of the fish fed each diet. Thus, the lower protein and higher lipid content of the HE diet group induced lower growth, increased liver size and elicited a higher percentage of muscle lipid when compared to the HP diet group. Long-term high dietary fat intake provokes a gradual deposition of lipids in several organs of this species, including the liver (Company et al., 1999). Moreover, the negative effect of high lipid diets on growth has been reported previously in gilthead seabream (Company et al., 1999), as well as in other species like dentex (Espinós et al., 2003) and sea bass (Peres and Oliva-Teles, 1999). However, improved growth occurred with the HE diet, meaning that no differences in growth were observed between the two diet groups when fish were subjected to sustained swimming in the present study. The HE group did maintain a significantly higher HSI, but this was probably due to increased fat deposition in this organ. Our group previously observed improved growth in seabream fingerlings fed a diet with a similar composition to that of the HP diet, but swimming at 5 BL/s^–1^ (Blasco et al., 2015). Furthermore, we previously reported a reduction in the lipid content of white muscle in exercised juveniles fed a high carbohydrate/low protein diet swimming at 1.5 BL/s^–1^ (Martin-Perez et al., 2012). These results indicated that gilthead seabream adapts their metabolism to the type of activity they are performing and their nutritional regime to prioritize the best fuel, as reviewed by Magnoni and coworkers (Magnoni et al., 2013b). The fuel selection depends on the type of exercise, with lipids the preferred fuel at low or medium velocities (i.e., aerobic training) and carbohydrates the preferred fuel as the velocity (and the energy demand) increases (Richards et al., 2002). The glycogen content of white muscle was reduced by the SS condition, regardless of the diet. These results corroborate the findings observed in the exercised juveniles of this species (Sánchez-Gurmaches et al., 2013), confirming that gilthead seabream subjected to exercise increases glucose oxidation to meet the high energy demands (Felip et al., 2013).

The isotopic composition of white muscle reveals clear differences in the effect of diet by reflecting the different proportions of vegetable-type components from a given feed (Beltrán et al., 2009; Martín-Pérez et al., 2011). Then, the δ^15^N values were lower in the HE diet group than in the HP diet group, both in the total muscle and in its protein fraction, reflecting the lower δ^15^N values of the HE diet probably due to the higher proportion of vegetable protein. The reduced ^13^C values in the lipid fraction of the HE diet group were due to the high lipid content in this tissue, which is in agreement with the findings of previous studies (Martín-Pérez et al., 2011; Martin-Pérez et al., 2013). Nitrogen dietary fractionation (Δδ^15^N) is a good marker of protein balance because it reflects the protein turnover and the protein retention efficiency (Martínez Del Rio et al., 2009; Martín-Pérez et al., 2011). Interestingly, fish fed the HP diet exhibited a lower fractionation than those fed the HE diet, indicating that less transformations of the protein from the diet (less protein turnover) were needed before being deposited into the muscle proteins (Gaye-Siessegger et al., 2003, 2007). The lower protein content of the HE diet with respect to the HP diet could contribute to this higher fractionation. In fact, a negative correlation between the protein percentage of the diet and nitrogen fractionation has been established for this species (Martin-Pérez et al., 2013). The lower growth of the HE diet group under VS conditions could reflect this higher fractionation, which is in agreement with that observed in gilthead seabream fed different proportions of plant proteins (Beltrán et al., 2009).

The SS condition did not modify the differences in isotopic composition of the diet or nitrogen fractionation of white muscle. Although the fractionation was different for each diet group under SS conditions, the final growth did not differ between the two groups and interaction was observed. This suggests different scenarios for each group. In the HE group, the greater use of dietary lipids for energy purposes might have induced a protein-sparing effect. In the HP diet group, the greater use of dietary protein for energy purposes might have resulted in less growth. This higher amino acid oxidation from the diet in the HP diet group under SS conditions is reflected in the higher nitrogen fractionation when compared to that under VS conditions, reducing the differences in this parameter between the two diet groups (VS: −0.46; SS: −0.34).

### Mitochondrial Adaptation in the Liver

As a key tissue in regulating metabolism, the liver presented changes in the gene expression patterns of mitochondrial proteins related to energy metabolism and fusion/fission balance in response to diet and exercise. In the present study, the significant decrease in the cox4/cs ratio in the HE group under VS conditions suggests that lipid oxidation was greater in this group. In agreement with this, LeMoine and coworkers (LeMoine et al., 2008) reported that the liver of goldfish fed a high-fat diet showed an increase in *cs* mRNA levels, but not in *cox* gene expression. Furthermore, Sánchez-Nuño and collegues (Sánchez-Nuño et al., 2018) demonstrated that gilthead seabream fed a high-fat diet showed an increase in the activity of CS, but not of COX. The COX/CS activity ratio indicates modifications of the mitochondrial surface/volume ratio (Ibarz et al., 2010; Martin-Perez et al., 2012), which reflects shifts in the use of nutrients. Thus, the significant decrease in the COX/CS ratio in the livers of fish fed a HE diet showed a clear metabolism shifting toward increased lipid oxidation in this group, which reflects that this tissue possesses a good plasticity for modifying the nutrient utilization depending on the diet composition. As a consequence of this shift, we observed increased reactive oxygen species (ROS) levels (data not shown, Sánchez-Moya et al., 2017), which could explain the increase in *ucp2* expression to uncouple oxidative phosphorylation. UCPs have a role as redox sensors that can serve to attenuate the effects of the ROS (Rial and Zardoya, 2009). The expression of *ucp1* increases when lipid flux to the liver increases in carp (Jastroch et al., 2005) and in gilthead seabream (Bermejo-Nogales et al., 2010). In our study, *ucp1* and *ucp2* showed different expression profiles, suggesting different roles of these proteins in achieving a balance between uncoupling activity and oxidative capacity. Although the role and tissue specificity of UCPs in gilthead seabream have been investigated, some overlap and redundancy might occur among UCPs, especially in relation to UCP2 (Bermejo-Nogales et al., 2010, 2014). High mRNA levels of *ucp2* and lower mRNA levels of *pgc1a* in the liver of the HE diet group under VS conditions are consistent with that observed in mammals presenting metabolic syndrome with increased lipid accumulation and ROS production (Rius-Pérez et al., 2020). By contrast, the higher mRNA levels of *pgc1a* in the HP diet group indicated a higher oxidation rate of dietary protein, yielding a lower energy output and explaining the lower expression of *ucp2.*

Under SS conditions, energy demands increased and the cox/cs ratio for mitochondrial energy proteins was affected with an interaction between diet and activity. Compared with VS conditions, a significant decrease in the cox/cs ratio, notably in the HP group, was associated with a reduced cox/cs ratio between the two diet groups. In other energy demanding conditions such as hypothermal stress, the COX/CS ratio has been reported to decrease, usually due to the increased expression and/or activity of CS (Lucassen et al., 2003; Eckerle et al., 2008; Ibarz et al., 2010; Orczewska et al., 2010; Sánchez-Nuño et al., 2018). Although swimming activity did not modify *ucp2* gene expression, no differences in *ucp2* expression were observed between the two diet groups, indicating a better balance between the TCA cycle and oxidative phosphorylation in the HE group under SS compared with VS conditions.

Diet induced changes in the expression of mitofusin, *mit 2*, and fission protein, *miffb*). No effect of swimming activity were observed in the expression of markers of mitochondrial dynamics. However, the significant interaction observed in the two mitofusins, *mit1* and *mit2* would indicate that the fusion processes are modulated by swimming activity depending of diet. In the present study, the simultaneous increase in the mRNA levels of *mit1*, *mit2* and *miffb* in the HE diet group under SS conditions indicated an increased mitochondrial turnover requiring both fusion and fission processes. This new balance represents an adaptation of the mitochondrial network in HE group toward a much higher oxidative capacity of the liver. In general, simultaneous mitochondrial fusion and fission processes indicate an improvement in the health status of a mitochondrial population (Scorrano, 2013), but the specific effects of exercise involve high expression of *mit1* and *mit2*, promoting the formation of a stronger network of healthy mitochondria (Liesa and Shirihai, 2013; Scorrano, 2013; Gonçalves et al., 2014, 2015). Interestingly, rats fed a high fat diet developed impaired mitochondrial function related to fatty deposition in liver, but endurance training was noted to prevent this pathological process (Gonçalves et al., 2014, 2015).

In mammals, it is postulated that mitofusins are directly regulated by *pgc1a* (Chandhok et al., 2018). We found that *pgc1a* expression was modulated by diet, being higher in the HP group and lower in the livers of fish in the HE group. The lower levels in the HE group may have indicated that this transcription factor was previously activated to promote *mit1*, *mit2*, and *miffb* upregulation before undergoing negative feedback. However, the close relationship between *pgc1a* and the downstream genes was not observed in the livers of fish under VS conditions, suggesting that *pgc1a* may not be the only regulator of these molecules in fish. The role of *pgc1a* in fish is currently under discussion [reviewed by Bremer and coworkers (Bremer et al., 2016)].

Thus, diet composition modified the liver oxidative capacity, because a higher content of lipids in the diet induced the uncoupling of the respiratory chain and decreased oxidation. Under SS conditions, uncoupling disappeared, lipid oxidation increased, and the mitochondria fusion process was promoted.

### Mitochondrial Adaptation in White and Red Skeletal Muscle

White muscle mitochondrial proteins related to energy metabolism (i.e., CS, UCP3, and PGC1a) were affected by diet. The higher lipid concentration in the muscle of fish fed a HE diet, together with the “oxidative overload,” modified the mitochondrial protein profile in comparison to the HP group, showing higher *cs, ucp3*, and lower *pgc1a* expressions. The higher oxidative state of white muscle would explain the increase in *ucp3* mRNA levels and its higher protein expression in this tissue, preventing the overproduction of ROS (Bermejo-Nogales et al., 2014). The differences between the gene and protein expression of PGC1a suggested that changes were already induced by the diet in the first weeks of feeding in the HE diet group. Thus, in the earlier weeks of the experiment, the high energy level of the HE diet might have triggered AMPK activation, which would then induce *pgc1a* expression to increase the oxidative capacity of the white muscle. The subsequent increase in the protein expression of PGC1a observed in the muscle would induce a strong negative feedback regulation on its gene expression, which is consistent with that observed in goldfish fed a high-fat diet (LeMoine et al., 2008). This regulation of *pgc1a* has been observed in mammals fed high-fat diets [in mice as reported by Bonnard and coworkers (Bonnard et al., 2008) and in humans as reported by Sparks and coworkers (Sparks et al., 2005)]. The lack of correlation between *cs* gene and CS protein expression illustrated the importance of post-transcriptional processes regulating this protein (Bustin et al., 2009).

Sustained moderate exercise induces a more aerobic phenotype in the white muscle of gilthead seabream (Martin-Perez et al., 2012; Blasco et al., 2015) and other species (Johnston and Moon, 1980; McClelland et al., 2006; Anttila et al., 2010), and this phenotype should be reflected by changes in the gene and protein expression of COX and CS enzymes. Swimming activity modified the expression of these metabolic proteins and related proteins (e.g., UCPs and PGC1a.) In this scenario of greater energy demand, the gene expression and protein expression of mitochondrial proteins associated with energy metabolism changed differently in white muscle depending on the diet (most showed interaction). The downregulation of cs expression in the HP group under SS conditions increased the cox/cs ratio when fed a HE diet; by contrast, reduced COX4 protein levels in the HE group decreased the COX/CS ratio. The significant decrease in *pgc1a* expression in HP group under the SS condition could be involved in *cs* downregulation. These results indicate that there is increased protein oxidation in HP group and increased lipid oxidation in the HE group. Interestingly, the increased *ucp3* expression found in the white muscle of fish fed a HE diet disappeared when shifting from VS to SS conditions. Activity appears to decrease the oxidative uncoupling associated with the HE diet and increases aerobic ATP production, consistent with observation in endurance trained humans (Russell et al., 2003; Schrauwen-Hinderling et al., 2003). There are no data about *ucp3* regulation in fish subjected to exercise, but in mammals, an inverse relationship has been observed between UCP3 and PGC1a, the latter being this a transcription factor stimulated by exercise in mammals but having an unclear role in fish (McClelland, 2012; Bremer et al., 2016). In zebrafish, a transient increase in PGC1a expression has been observed to occur at the beginning of exercise (LeMoine et al., 2010). It needs to be demonstrated whether such a response occurred in the first days of exercise in gilthead seabream. Diet did not modify the gene expression of fusion/fission proteins, but activity decreased the expression of *mit2* in both diet groups and of the *fis1* and *miffb* (related the fission processes), changing the balance between the fusion and fission processes in the VS condition. A significant reduction in *fis1* gene expression due to activity in HE group could indicate that the mitochondrial machinery works more efficiently with a higher lipid content during exercise. In mammals, lipid accumulation disrupts mitochondrial homeostasis (Lowell and Shulman, 2005), and is accompanied by a greater increase in fission compared to fusion processes in skeletal muscle (Holmström et al., 2012; Huertas et al., 2019). This increases mitochondrial degradation by mitophagy, promoting mitochondrial turnover (Chandhok et al., 2018).

It is interesting to note how the expression profile of genes and mitochondrial proteins in red muscle differed from that of white muscle between the two diet groups. These differences would be due to the nutrients that the muscles prefer to use to obtain energy, with red muscle preferring lipids and white muscle preferring carbohydrates (Magnoni et al., 2013b). Moreover, red muscle is an oxidative tissue with a high proportion of mitochondria. COX4 and CS activities are 3 and 7 times higher, respectively, in red muscle than in white muscle in gilthead seabream (Martin-Perez et al., 2012). Diet modified *cs* expression, and consequently the *cox/cs* ratio, as well as *pgc1a* expression, with *cs* and *pgc*1a expression being lower in HE group. The higher protein expression of CS and COX in this group would indicate that the high lipid content of HE diet did not exceed the oxidative capacity of this tissue. The downregulation of the *cs* gene expression might have occurred as a response to the high increase of its protein expression. Moreover, the significantly higher COX/CS ratio, both at the gene and protein level in HE diet group, indicated increased lipid oxidation in this tissue. In contrast to white muscle, red muscle has a lipid level that is 10 times higher in this species (Martin-Perez et al., 2012). In red muscle, which is adapted to lipid oxidation, no changes in the expression of *ucp3* gene or its protein induced by diet were observed. This indicated that the high lipid content of HE diet not exceed the oxidative capacity of this tissue.

The activity did not provoke changes in the expression of these genes, *cs* and *cox*. But the increase in UCP3 levels in the red muscle of the HE group under SS conditions suggests that these enzymes are regulated to adapt to a high lipid overload, such as that observed in the skeletal and cardiac muscle of mammals (Nabben et al., 2014). The impact of swimming on mitochondrial proteins related to energy metabolism was greater in the white muscle compared with the red muscle of gilthead seabream. It agrees with a previous study in our group showing that the red muscle workload under sustained exercise is reduced, but that increased white muscle activity compensates for this (Martin-Perez et al., 2012). It is interesting to know that the composition of the diet modifies more than the activity, the balance between the processes of fusion and fission, but more research is needed to understand the meaning of the changes.

The composition of the diet affected the oxidative capacities of both white and red muscles, increasing their oxidative rates when fish were fed a higher lipid content, especially in the red muscle. Increased lipid oxidation eventually caused uncoupling in white muscle, but not in red muscle. The SS condition was able to reverse these changes, promoting the uncoupling in red muscle and eliminating it in white muscle. In this sense, the decrease in the *fis1* gene expression in white muscle would indicate the improvement of mitochondrial health by moderate, sustained exercise.

### Conclusion

In this study, we show how changes in diet composition (lower protein/lipid ratio) and average physical activity (voluntary swimming versus sustained exercise) affected the body growth of juvenile sea bream differently. Lower protein/lipid ratio affected growth through unbalanced availability and use of nutrients, especially indicated by changes in key mitochondrial proteins related to energy metabolism, mitochondrial turnover, and biogenesis in the liver and white and red and white muscles. The beneficial effects of sustained swimming reversed the altered mitochondrial functions by increasing the use of lipids, along with a protein sparing effect of enhanced growth. From an applied point of view, a cheaper (reducing the protein/lipid ratio) and sustainable (reducing nitrogenous waste) diet can be used in combination with moderate swimming to preserve fish growth, even in the period of fast growth of juvenile sea bream, and to improve the welfare of fish.

## Data Availability Statement

The original contributions presented in the study are included in the article/Supplementary Material, further inquiries can be directed to the corresponding author.

## Ethics Statement

The animal study was reviewed and approved by Comité Ético de Experimentación Animal de la Universidad de Barcelona (CEEA 663/13 and DAAM 7644).

## Author Contributions

JB, JF-B, and JG conceived and designed the study. MP-A, EV, AS-M, and IG-P performed the experiment and laboratory analysis. MP-A and JB analyzed the data and drafted the manuscript. All authors contributed to the writing and approved the submitted version of the manuscript.

## Conflict of Interest

The authors declare that the research was conducted in the absence of any commercial or financial relationships that could be construed as a potential conflict of interest.

## References

[B1] Anttila K. Jäntti M. Mänttäri S. (2010). Effects of training on lipid metabolism in swimming muscles of sea trout E V ects of training on lipid metabolism in swimming muscles of sea trout (*Salmo trutta*). *J. Comp. Physiol. B* 180 707–714. 10.1007/s00360-010-0446-1 20135129

[B2] Baar K. Wende A. R. Jones T. E. Marison M. Nolte L. A. Chen M. A. Y. (2002). Adaptations of skeletal muscle to exercise: rapid increase in the transcriptional coactivator PGC-1. *FASEB J.* 1 1879–1886. 10.1096/fj.02-0367com 12468452

[B3] Beltrán M. Fernández-Borrás J. Médale F. Pérez-Sánchez J. Kaushik S. Blasco J. (2009). Natural abundance of 15N and 13C in fish tissues and the use of stable isotopes as dietary protein tracers in rainbow trout and gilthead sea bream. *Aquac. Nutr.* 15 9–18. 10.1111/j.1365-2095.2008.00563.x

[B4] Bermejo-Nogales A. Benedito-Palos L. Calduch-Giner J. A. Pérez-Sánchez J. (2011). Feed restriction up-regulates uncoupling protein 3 (UCP3) gene expression in heart and red muscle tissues of gilthead sea bream (*Sparus aurata* L.). New insights in substrate oxidation and energy expenditure. *Comp. Biochem. Physiol. A Mol. Integr. Physiol.* 159 296–302. 10.1016/j.cbpa.2011.03.024 21463702

[B5] Bermejo-Nogales A. Calduch-Giner J. A. Pérez-Sánchez J. (2010). Gene expression survey of mitochondrial uncoupling proteins (UCP1/UCP3) in gilthead sea bream (*Sparus aurata* L.). *J. Comp. Physiol. B Biochem. Syst. Environ. Physiol.* 180 685–694. 10.1007/s00360-009-0441-6 20063001

[B6] Bermejo-Nogales A. Calduch-Giner J. A. Pérez-Sánchez J. (2014). Tissue-specific gene expression and functional regulation of uncoupling protein 2 (UCP2) by hypoxia and nutrient availability in gilthead sea bream (*Sparus aurata*): implications on the physiological significance of UCP1-3 variants. *Fish Physiol. Biochem.* 40 751–762. 10.1007/s10695-013-9882-7 24154671

[B7] Blasco J. Moya A. Millán-Cubillo A. Vélez E. J. Capilla E. Pérez-Sánchez J. (2015). Growth-promoting effects of sustained swimming in fingerlings of gilthead sea bream (*Sparus aurata* L.). *J. Comp. Physiol. B Biochem. Syst. Environ. Physiol.* 185 859–868. 10.1007/s00360-015-0933-5 26391594

[B8] Bligh E. G. Dyer W. J. (1959). A rapid method of total lipid extraction and purification. *Can. J. Biochem. Physiol.* 37 911–917. 10.1139/o59-099 13671378

[B9] Bonnard C. Durand A. Peyrol S. Chanseaume E. Chauvin M.-A. Morlo B. (2008). Mitochondrial dysfunction results from oxidative stress in the skeletal muscle of diet-induced insulin-resistant mice. *J. Clin. Invest.* 118 789–800. 10.1172/JCI32601 18188455 PMC2176186

[B10] Bremer K. Kocha K. M. Snider T. Moyes C. D. (2016). Sensing and responding to energetic stress: The role of the AMPK-PGC1α-NRF1 axis in control of mitochondrial biogenesis in fish. *Comp. Biochem. Physiol. Part B Biochem. Mol. Biol.* 199 4–12. 10.1016/j.cbpb.2015.09.005 26393435

[B11] Bustin S. A. Benes V. Garson J. A. Hellemans J. Huggett J. Kubista M. (2009). The MIQE guidelines: minimum information for publication of quantitative real-time PCR experiments. *Clin. Chem.* 55 611–622. 10.1373/clinchem.2008.112797 19246619

[B12] Caballero M. J. López-Calero G. Socorro J. Roo F. J. Izquierdo M. S. Férnandez A. J. (1999). Combined effect of lipid level and fish meal quality on liver histology of gilthead seabream (*Sparus aurata*). *Aquaculture* 179 277–290. 10.1016/S0044-8486(99)00165-9

[B13] Chandhok G. Lazarou M. Neumann B. (2018). Structure, function, and regulation of mitofusin-2 in health and disease. *Biol. Rev.* 93 933–949. 10.1111/brv.12378 29068134 PMC6446723

[B14] Chen P. B. Yang J. S. Park Y. (2018). Adaptations of Skeletal muscle mitochondria to obesity, exercise, and polyunsaturated fatty acids. *Lipids* 53 271–278. 10.1002/lipd.12037 29663395

[B15] Company R. Calduch-Giner J. A. Kaushik S. Pérez-Sánchez J. (1999). Growth performance and adiposity in gilthead sea bream (*Sparus aurata*): Risks and benefits of high energy diets. *Aquaculture* 171 279–292. 10.1016/S0044-8486(98)00495-5

[B16] Coulibaly I. Gahr S. A. Palti Y. Yao J. Rexroad C. E. (2006). Genomic structure and expression of uncoupling protein 2 genes in rainbow trout (*Oncorhynchus mykiss*). *BMC Genomics* 7:203. 10.1186/1471-2164-7-203 16899121 PMC1559616

[B17] Davison W. (1997). The effects of exercise training on teleost fish, a review of recent literature. *Comp. Biochem. Physiol. Part A Physiol.* 117 67–75. 10.1016/S0300-9629(96)00284-8

[B18] Diano S. Horvath T. L. (2012). Mitochondrial uncoupling protein 2 (UCP2) in glucose and lipid metabolism. *Trends Mol. Med.* 18 52–58. 10.1016/j.molmed.2011.08.003 21917523

[B19] Divakaruni A. S. Brand M. D. (2011). The regulation and physiology of mitochondrial proton leak. *Physiology* 26 192–205. 10.1152/physiol.00046.2010 21670165

[B20] Eckerle L. G. Lucassen M. Hirse T. Pörtner H. O. (2008). Cold induced changes of adenosine levels in common eelpout (*Zoarces viviparus*): A role in modulating cytochrome c oxidase expression. *J. Exp Biol.* 211 1262–1269. 10.1242/jeb.013474 18375851

[B21] Espinós F. J. Tomás A. Pérez L. M. Balasch S. Jover M. (2003). Growth of dentex fingerlings (*Dentex dentex*) fed diets containing different levels of protein and lipid. *Aquaculture* 218 479–490. 10.1016/S0044-8486(02)00313-7

[B22] Eya J. C. Ashame M. F. Pomeroy C. F. Manning B. B. Peterson B. C. (2012). Genetic variation in feed consumption, growth, nutrient utilization efficiency and mitochondrial function within a farmed population of channel catfish (*Ictalurus punctatus*). *Comp. Biochem. Physiol. Part B.* 163 211–220. 10.1016/j.cbpb.2012.05.019 22691874

[B23] Eya J. C. Yossa R. Perera D. Okubajo O. Gannam A. (2017). Combined effects of diets and temperature on mitochondrial function, growth and nutrient efficiency in rainbow trout (*Oncorhynchus mykiss*). *Comp. Biochem. Physiol. Part B.* 212 1–11. 10.1016/j.cbpb.2017.06.010 28687361

[B24] Felip O. Blasco J. Ibarz A. Martin-Pérez M. Fernández-Borràs J. (2013). Beneficial effects of sustained activity on the use of dietary protein and carbohydrate traced with stable isotopes 15N and 13C in gilthead sea bream (*Sparus aurata*). *J. Comp. Physiol. B Biochem. Syst. Environ. Physiol.* 183 223–234. 10.1007/s00360-012-0703-6 22918602

[B25] Felip O. Ibarz A. Fernández-Borràs J. Beltrán M. Martín-Pérez M. Planas J. V. (2012). Tracing metabolic routes of dietary carbohydrate and protein in rainbow trout (*Oncorhynchus mykiss*) using stable isotopes ([13 C] starch and [15 N] protein): effects of gelatinisation of starches and sustained swimming. *Br. J. Nutr.* 107 834–844. 10.1017/S0007114511003709 21806854

[B26] Fraga F. (1956). Determinación de glucógeno en moluscos con el reactivo de antrona. *Inv. Pesq.* 3 69–74.

[B27] Gannes L. Z. Marti C. (1998). Natural abundance variations in stable isotopes and their potential uses in animal physiological ecology. *Comp. Biochem. Physiol. Part A.* 119 725–737. 10.1016/S1095-6433(98)01016-2 9683412

[B28] Gaye-Siessegger J. Focken U. Abel H. Becker K. (2007). Influence of dietary non-essential amino acid profile on growth performance and amino acid metabolism of *Nile tilapia*, *Oreochromis niloticus* (L.). *Comp. Biochem. Physiol. A Mol. Integr. Physiol.* 146 71–77. 10.1016/j.cbpa.2006.09.025 17157045

[B29] Gaye-Siessegger J. Focken U. Abel H.-J. Becker K. (2003). Feeding level and diet quality influence trophic shift of c and n isotopes in *Nile tilapia* (*Oreochromis niloticus* (L.)). *Isotopes Environ Health Stud.* 39 125–134. 10.1080/1025601031000113556 12872804

[B30] Gonçalves I. O. Passos E. Diogo C. V. Rocha-Rodrigues S. Santos-Alves E. Oliveira P. J. (2015). Exercise mitigates mitochondrial permeability transition pore and quality control mechanisms alterations in nonalcoholic steatohepatitis. *Appl. Physiol. Nutr. Metab.* 41 298–306. 10.1139/apnm-2015-0470 26905378

[B31] Gonçalves I. O. Passos E. Rocha-Rodrigues S. Diogo C. V. Torrella J. R. Rizo D. (2014). Physical exercise prevents and mitigates non-alcoholic steatohepatitis-induced liver mitochondrial structural and bioenergetics impairments. *Mitochondrion* 15 40–51. 10.1016/j.mito.2014.03.012 24727595

[B32] Good C. A. Kramer H. Somogyi M. (1933). The determination of glycogen. *J. Biol. Chem.* 100 485–491. 10.1016/S0021-9258(18)75966-8

[B33] Holmström M. H. Iglesias-Gutierrez E. Zierath J. R. Garcia-Roves P. M. (2012). Tissue-specific control of mitochondrial respiration in obesity-related insulin resistance and diabetes. *Am. J. Physiol. Endocrinol. Metab.* 302 731–739. 10.1152/ajpendo.00159.2011 22252943

[B34] Huertas J. R. Casuso R. A. Agustín P. H. Cogliati S. (2019). Stay fit, stay young: mitochondria in movement: the role of exercise in the new mitochondrial paradigm. *Oxid. Med. Cell Longev* 2019:7058350. 10.1155/2019/7058350 31320983 PMC6607712

[B35] Ibarz A. Blasco J. Gallardo M. A. Fernández-Borràs J. (2010). Energy reserves and metabolic status affect the acclimation of gilthead sea bream (*Sparus aurata*) to cold. *Comp. Biochem. Physiol. A Mol. Integr. Physiol.* 155 319–326. 10.1016/j.cbpa.2009.11.012 19931633

[B36] Ibarz A. Felip O. Fernández-Borràs J. Martín-Pérez M. Blasco J. Torrella J. R. (2011). Sustained swimming improves muscle growth and cellularity in gilthead sea bream. *J. Comp. Physiol. B Biochem. Syst. Environ. Physiol.* 181 209–217. 10.1007/s00360-010-0516-4 20882387

[B37] Jastroch M. Wuertz S. Kloas W. Klingenspor M. (2005). Uncoupling protein 1 in fish uncovers an ancient evolutionary history of mammalian nonshivering thermogenesis. *Physiol. Genom.* 22 150–156. 10.1152/physiolgenomics.00070.2005 15886331

[B38] Johnston I. A. Moon T. W. (1980). Exercise training in skeletal muscle of brook trout (*Salvelinus fontinalis*). *J. Exp. Biol.* 87 177–194. 10.1242/jeb.87.1.177 7420013

[B39] Leaver M. J. Bautista J. M. Björnsson B. T. Jönsson E. Krey G. Tocher D. R. (2008). Towards fish lipid nutrigenomics: current state and prospects for fin-fish aquaculture. *Rev. Fish Sci.* 16(Suppl 1) 71–92. 10.1080/10641260802325278

[B40] LeMoine C. M. R. Craig P. M. Dhekney K. Kim J. J. McClelland G. B. (2010). Temporal and spatial patterns of gene expression in skeletal muscles in response to swim training in adult zebrafish (*Danio rerio*). *J. Comp. Physiol. B Biochem. Syst. Environ. Physiol.* 180 151–160. 10.1007/s00360-009-0398-5 19693513

[B41] LeMoine C. M. R. Genge C. E. Moyes C. D. (2008). Role of the PGC-1 family in the metabolic adaptation of goldfish to diet and temperature. *J. Exp. Biol.* 211 1448–1455. 10.1242/jeb.014951 18424678

[B42] Li X. Wang B. Xu C. Shi H. Zhang L. Liu J. (2019). Regulation of mitochondrial biosynthesis and function by dietary carbohydrate levels and lipid sources in juvenile blunt snout bream *Megalobrama amblycephala*. *Comp. Biochem. Physiol. Part A.* 227 14–24. 10.1016/j.cbpa.2018.08.008 30201543

[B43] Liesa M. Shirihai O. S. (2013). Mitochondrial dynamics in the regulation of nutrient utilization and energy expenditure. *Cell Metab.* 17 491–506. 10.1016/j.cmet.2013.03.002 23562075 PMC5967396

[B44] Lowell B. B. Shulman G. I. (2005). Mitochondrial dysfunction and type 2 diabetes. *Curr. Diab. Rep.* 5 177–183. 10.1126/science.1104343 15929863 PMC2995500

[B45] Lucassen M. Schmidt A. Eckerle L. G. Pörtner H. O. (2003). Mitochondrial proliferation in the permanent vs. temporary cold: enzyme activities and mRNA levels in Antarctic and temperate zoarcid fish. *Am. J. Physiol. Regul. Integr. Comp. Physiol.* 285 1410–1420. 10.1152/ajpregu.00111.2003 12907412

[B46] Magnoni L. J. Crespo D. Ibarz A. Blasco J. Fernández-Borràs J. Planas J. V. (2013a). Effects of sustained swimming on the red and white muscle transcriptome of rainbow trout (*Oncorhynchus mykiss*) fed a carbohydrate-rich diet. *Comp. Biochem. Physiol. A Mol. Integr. Physiol.* 166 510–521. 10.1016/j.cbpa.2013.08.005 23968867

[B47] Magnoni L. J. Felip O. Blasco J. Planas J. V. (2013b). “Metabolic Fuel utilization during swimming: optimizing nutritional requirements for enhanced performance,” in *Swimming Physiology of Fish: Towards Using Exercise to Farm a Fit Fish in Sustainable Aquaculture*, eds Palstra A. P. Planas J. V. (Berlin, Heidelberg: Springer Berlin Heidelberg), 203–235. 10.1007/978-3-642-31049-2_9

[B48] Martínez Del Rio C. Wolf N. Carleton S. A. Gannes L. Z. (2009). Isotopic ecology ten years after a call for more laboratory experiments. *Biol. Rev.* 84 91–111. 10.1111/j.1469-185X.2008.00064 19046398

[B49] Martin-Pérez M. Fernández-Borràs J. Ibarz A. Felip O. Fontanillas R. Gutiérrez J. (2013). Naturally occurring stable isotopes reflect changes in protein turnover and growth in gilthead sea bream (*Sparus aurata*) juveniles under different dietary protein levels. *J. Agric. Food Chem.* 61 8924–8933. 10.1021/jf402617h 23947425

[B50] Martín-Pérez M. Fernández-Borràs J. Ibarz A. Felip O. Gutiérrez J. Blasco J. (2011). Stable isotope analysis combined with metabolic indices discriminates between gilthead sea bream (*Sparus aurata*) fingerlings produced in various hatcheries. *J. Agric. Food Chem.* 59:11893. 10.1021/jf201670t 21838305

[B51] Martin-Perez M. Fernandez-Borras J. Ibarz A. Millan-Cubillo A. Felip O. De Oliveira E. (2012). New insights into fish swimming: a proteomic and isotopic approach in gilthead sea bream. *J. Proteome Res.* 11 3533–3547. 22681184 10.1021/pr3002832

[B52] McClelland G. B. (2012). Muscle remodeling and the exercise physiology of fish. *Exerc. Sport Sci. Rev.* 40 165–173. 10.1097/JES.0b013e3182571e2c 22732426

[B53] McClelland G. B. Craig P. M. Dhekney K. Dipardo S. (2006). Temperature- and exercise-induced gene expression and metabolic enzyme changes in skeletal muscle of adult zebrafish (*Danio rerio*). *J. Physiol.* 577 739–751. 10.1113/jphysiol.2006.119032 16990399 PMC1890438

[B54] McClelland G. B. Scott G. R. (2014). “Muscle plasticity,” in *The Physiology of Fishes*, 4th Edn, eds Evans D. H. Claiborne J. B. Currie S. (Boca Ratón, FL: CRC Press), 1–31. 10.1007/1-4020-5177-8_1

[B55] McKenzie D. J. Palstra A. P. Planas J. Mackenzie S. Thorarensen H. Vandeputte M. (2020). Aerobic swimming in intensive finfish aquaculture: applications for production, mitigation and selection. *Rev. Aquacul.* 13 138–155. 10.1111/raq.12467

[B56] Meyer J. N. Leuthner T. C. Luz A. L. (2017). Mitochondrial fusion, fission, and mitochondrial toxicity. *Toxicology* 391 42–53. 10.1016/j.tox.2017.07.019 28789970 PMC5681418

[B57] Milder J. B. Petel M. (2012). Modulation of oxidative stress and mitochondrial function by the ketogenic diet. *Epilepsy Res.* 100 295–303. 10.1016/j.eplepsyres.2011.09.021 22078747 PMC3322307

[B58] Nabben M. van Bree B. W. J. Lenaers E. Hoeks J. Hesselink M. K. C. Schaart G. (2014). Lack of UCP3 does not affect skeletal muscle mitochondrial function under lipid-challenged conditions, but leads to sudden cardiac death. *Basic Res. Cardiol.* 109:447. 10.1007/s00395-014-0447-4 25344084 PMC4329241

[B59] Ni H. M. Williams J. A. Ding W. X. (2015). Mitochondrial dynamics and mitochondrial quality control. *Redox Biol.* 4 6–13. 10.1016/j.redox.2014.11.006 25479550 PMC4309858

[B60] Orczewska J. I. Hartleben G. O’Brien K. M. (2010). The molecular basis of aerobic metabolic remodeling differs between oxidative muscle and liver of threespine sticklebacks in response to cold acclimation. *Am. J. Physiol. Regul. Integr. Comp. Physiol.* 299 352–364. 10.1152/ajpregu.00189.2010 20427717

[B61] Palstra A. P. Kals J. Böhm T. Bastiaansen J. W. M. (2020). Swimming performance and oxygen consumption as non-lethal indicators of production traits in Atlantic salmon and gilthead seabream. *Front. Physiol.* 11:759. 10.3389/fphys.2020.00759 32733272 PMC7358457

[B62] Palstra A. P. Planas J. V. (2013). *Swimming Physiology of Fish: Towards Using Exercise to Farm a Fit Fish in Sustainable Aquaculture. Swim Physiol Fish Towards Using Exerc to Farm a Fit Fish Sustainable Aquaculture.* New York, NY: Springer-Verlag Berlin Heidelberg, 430.

[B63] Peres H. Oliva-Teles A. (1999). Effect of dietary lipid level on growth performance and feed utilization by European sea bass juveniles (*Dicentrarchus labrax*). *Aquaculture* 179 325–334. 10.1016/S0044-8486(99)00168-4

[B64] Pfaffl M. W. (2001). A new mathematical model for relative quantification in real-time RT-PCR. *Nucleic Acids Res.* 29:e45. 10.1093/nar/29.9.e45 11328886 PMC55695

[B65] Puigserver P. Wu Z. Park C. W. Graves R. Wright M. Spiegelman B. M. A. (1998). Cold-inducible coactivator of nuclear receptors linked to adaptive thermogenesis. *Cell* 92 829–839. 10.1016/S0092-8674(00)81410-5 9529258

[B66] Rial E. Zardoya R. (2009). Oxidative stress, thermogenesis and evolution of uncoupling proteins. *J. Biol.* 8 2–6. 10.1186/jbiol155 19589183 PMC2737370

[B67] Richards J. G. Mercado A. J. Clayton C. A. Heigenhauser G. J. F. Wood C. M. (2002). Substrate utilization during graded aerobic exercise in rainbow trout. *J. Exp. Biol.* 2077 2067–2077. 10.1242/jeb.205.14.2067 12089210

[B68] Rius-Pérez S. Torres-cuevas I. Millán I. Ortega ÁL. Pérez S. (2020). PGC-1α, inflammation, and oxidative stress: an integrative view in metabolism. *Oxid Med. Cell Longev* 2020:1452696. 10.1155/2020/1452696 32215168 PMC7085407

[B69] Russell A. P. Somm E. Praz M. Crettenand A. Hartley O. Melotti A. (2003). UCP3 protein regulation in human skeletal muscle fibre types I, IIa and IIx is dependent on exercise intensity. *J. Physiol.* 550 855–861. 10.1113/jphysiol.2003.040162 12794174 PMC2343085

[B70] Sánchez-Gurmaches J. Cruz-Garcia L. Ibarz A. Fernández-Borrás J. Blasco J. Gutiérrez J. (2013). Insulin, IGF-I, and muscle MAPK pathway responses after sustained exercise and their contribution to growth and lipid metabolism regulation in gilthead sea bream. *Domest. Anim. Endocrinol.* 45 145–153. 10.1016/j.domaniend.2013.08.001 24011532

[B71] Sánchez-Moya A. Viñuales J. Véliz R. Vélez E. J. Perelló M. Gutiérrez J. (2017). “Combined effect of diet and exercise on protective metabolism in gilthead sea bream (*Sparus aurata*),” in *Proceedings of the 16th National Congress of Aquaculture (Spanish Aquaculture Society)*, Zaragoza.

[B72] Sánchez-Nuño S. Eroldogan O. T. Sanahuja I. Blasco J. Fernández-borràs J. Fontanillas R. (2018). Cold-induced growth arrest in gilthead sea bream Sparus aurata: metabolic reorganisation and recovery. *Aquacult. Environ. Interact.* 10 511–528. 10.3354/aei00286

[B73] Scarpulla R. C. (2011). Metabolic control of mitochondrial biogenesis through the PGC-1 family regulatory network. *Biochim. Biophys. Acta Mol. Cell Res.* 1813 1269–1278. 10.1016/j.bbamcr.2010.09.019 20933024 PMC3035754

[B74] Schrauwen-Hinderling V. B. Schrauwen P. Hesselink M. K. C. Van Engelshoven J. M. A. Nicolay K. Saris W. H. M. (2003). The increase in intramyocellular lipid content is a very early response to training. *J. Clin. Endocrinol. Metab.* 88 1610–1616. 10.1210/jc.2002-021464 12679446

[B75] Scorrano L. (2013). Keeping mitochondria in shape: a matter of life and death. *Eur. J. Clin. Invest.* 43 886–893. 10.1111/eci.12135 23869410

[B76] Smith R. L. Soeters M. R. Wüst R. C. I. Houtkooper R. H. (2018). Metabolic flexibility as an adaptation to energy resources and requirements in health and disease. *Endocr. Rev.* 39 489–517. 10.1210/er.2017-00211 29697773 PMC6093334

[B77] Song Y. Hogstrand C. Ling S. Chen G. Luo Z. (2020). Creb-Pgc1 α pathway modulates the interaction between lipid droplets and mitochondria and influences high fat diet-induced changes of lipid metabolism in the liver and isolated hepatocytes of yellow catfish. *J. Nutr. Biochem.* 80:108364. 10.1016/j.jnutbio.2020.108364 32199344

[B78] Sparks L. M. Xie H. Koza R. A. Mynatt R. Hulver M. W. Bray G. A. (2005). A high-fat diet coordinately downregulates genes required for mitochondrial oxidative phosphorylation in skeletal muscle. *Diabetes* 54 1926–1933. 10.2337/diabetes.54.7.1926 15983191

[B79] Vélez E. J. Azizi S. Millán-Cubillo A. Fernández-Borràs J. Blasco J. Chan S. J. (2016). Effects of sustained exercise on GH-IGFs axis in gilthead sea bream (*Sparus aurata*). *Am. J. Physiol. Regul. Integr. Comp. Physiol.* 310 R313–R322. 10.1152/ajpregu.00230.2015 26661095

[B80] Westermann B. (2010). Mitochondrial fusion and fission in cell life and death. *Nat. Rev. Mol. Cell Biol.* 11 872–884. 10.1038/nrm3013 21102612

[B81] Westermann B. (2012). Bioenergetic role of mitochondrial fusion and fission. *Biochim. Biophys. Acta Bioenergy* 1817 1833–1838. 10.1016/j.bbabio.2012.02.033 22409868

[B82] Wu Z. Puigserver P. Andersson U. Zhang C. Adelmant G. Mootha V. (1999). Mechanisms controlling mitochondrial biogenesis and respiration through the thermogenic coactivator PGC-1. *Cell* 98 115–124. 10.1016/S0092-8674(00)80611-X 10412986

