# Supplementary material for: Mitochondrial Adaptation to Diet and Swimming Activity in Gilthead Seabream: Improved Nutritional Efficiency
Source: Front Physiol. 2021 Jun 18;12:678985. doi: 10.3389/fphys.2021.678985 (PMC8249818; doi:10.3389/fphys.2021.678985)

Supplementary Material

1 Supplementary Data

The supplementary material includes the original images of the Western blots and their respective total protein stains used in the present work. For each tissue, skeletal white muscle (WM) and skeletal red muscle (RM), two gels were performed for each protein and tissue, and consequently, a total of six samples per protein and tissue were used to calculate the results. The distribution of the samples within each gel is the same for all of them (see description in the COX and CS images from WM samples), where wells 1-3 correspond to VS-HE; 4-6 to SS-HP; 7-9 to VS-HP and 10-12 to SS-HE. Note that for all the proteins except the UCP3 in WM, all the gels present 13 wells. In those gels, the first well from the left of each gel (marked as LC) was a sample used as an intermembrane loading control. In the case of the COX4 and the CS, the Western blots were prepared from the same membranes, which were split after the transfer in order to incubate each piece with the corresponding antibodies. For the preparation of the figures, sets of two consecutive representative bands from both the blots and the corresponding total protein stains were cropped.

COX4 and CS

WM

MEMB 1

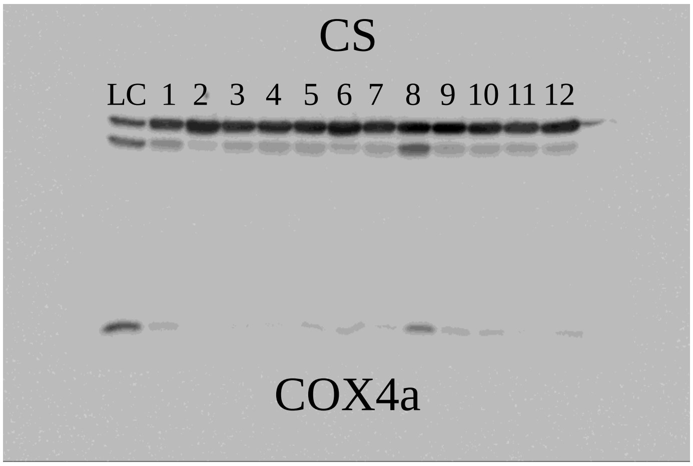

MEMB 2

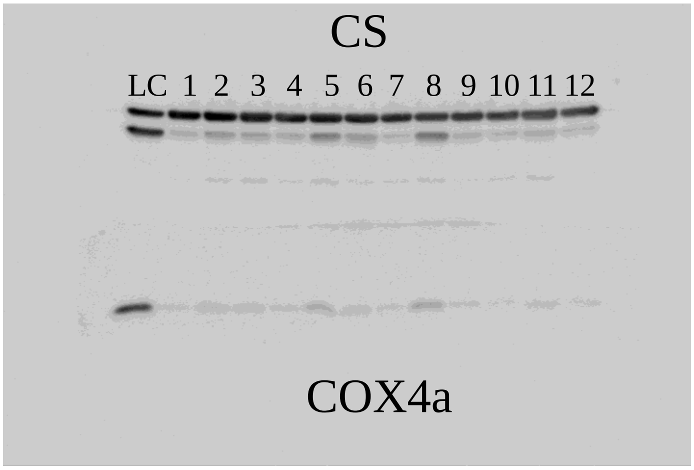

MEMB 1 TOTAL PROTEIN

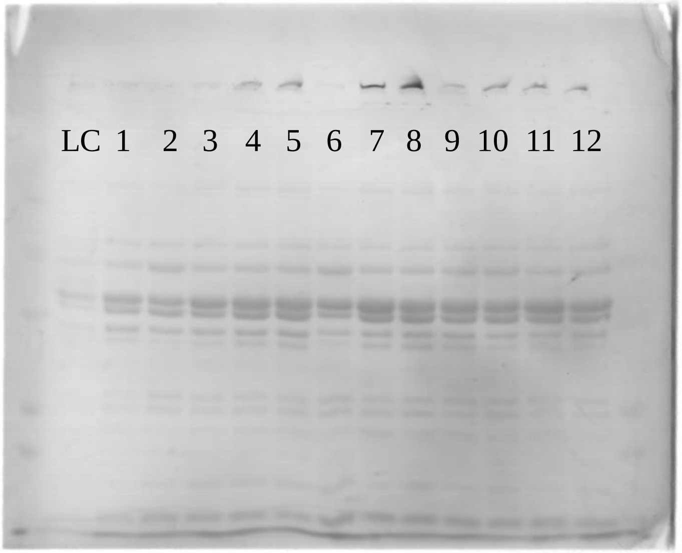

MEMB 2 TOTAL PROTEIN

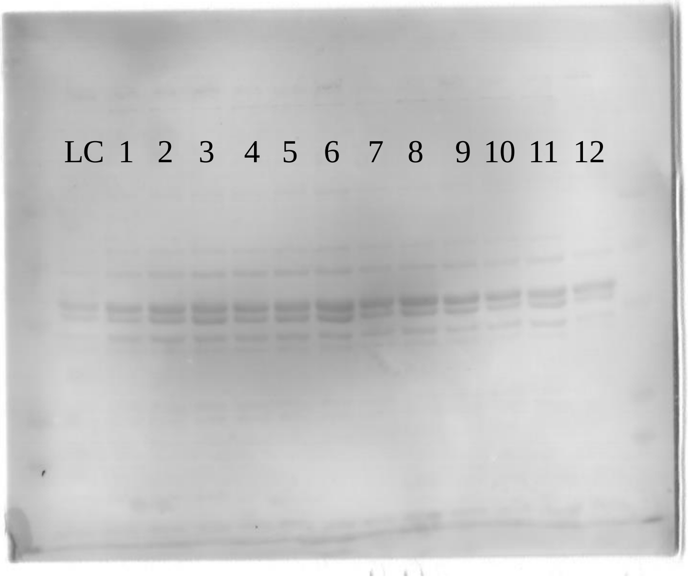

COX4 and CS

RM

MEMB 3

MEMB 4

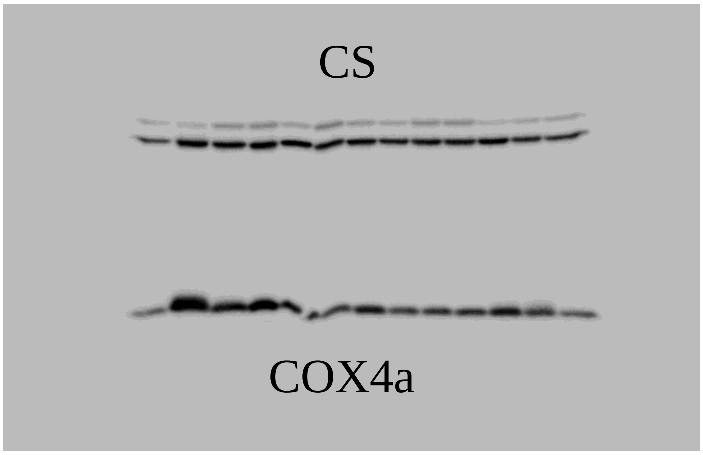

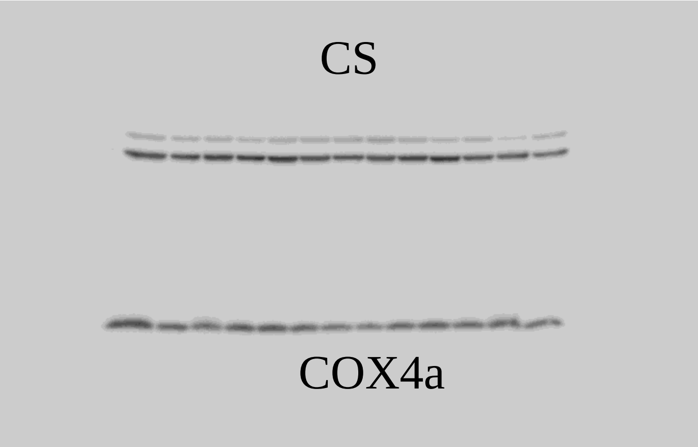

MEMB 3 TOTAL PROTEIN

MEMB 3 TOTAL PROTEIN

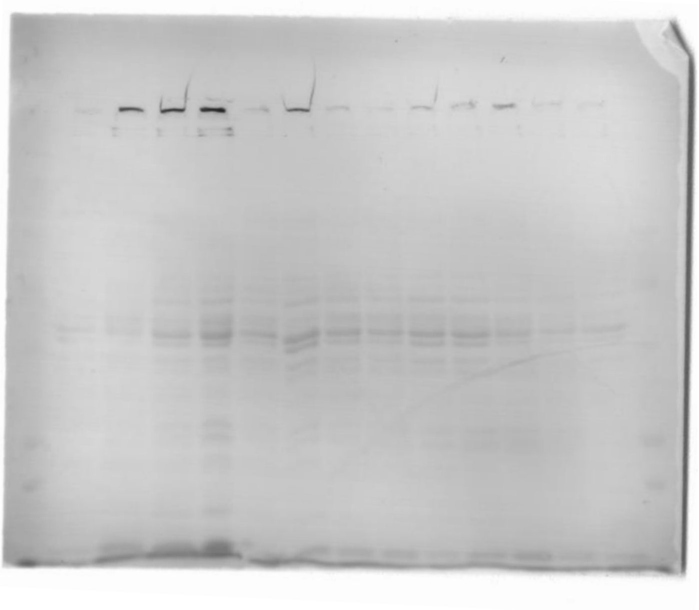

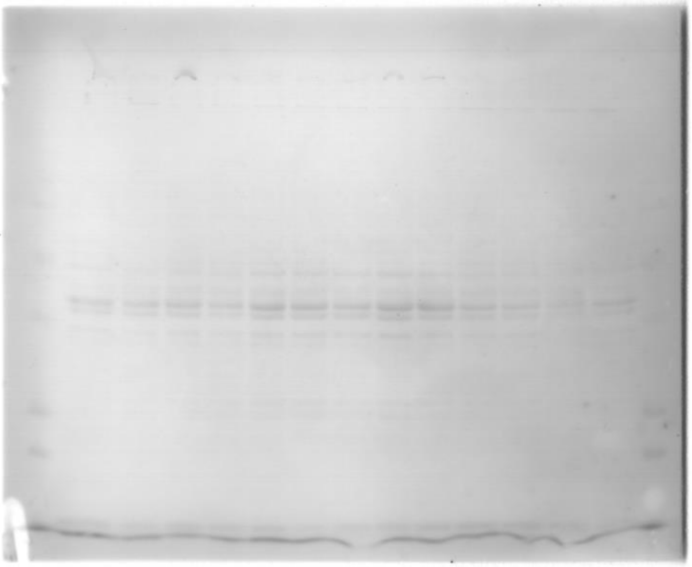

PGC1a

WM

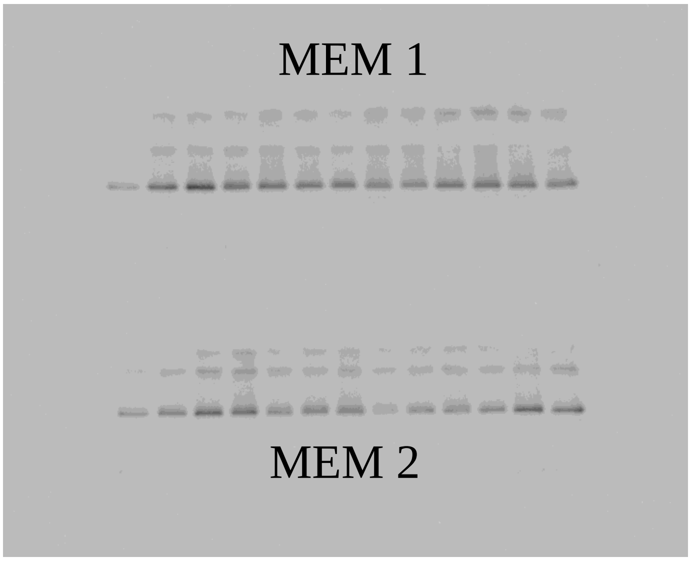

RM

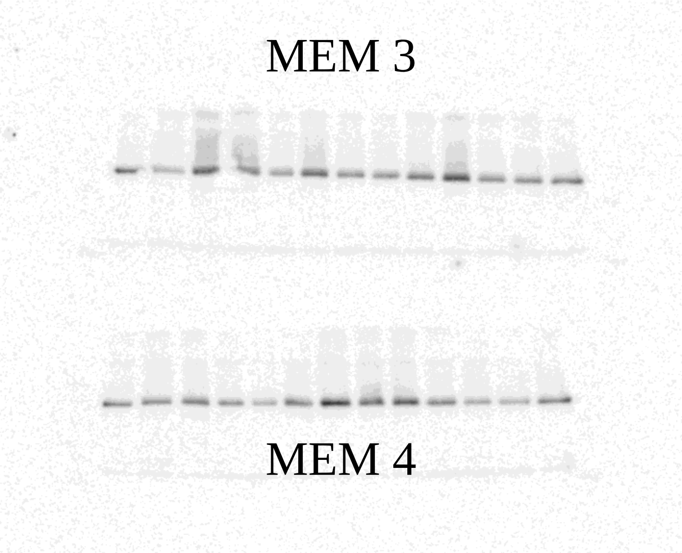

MEM 1 TOTAL PROTEIN

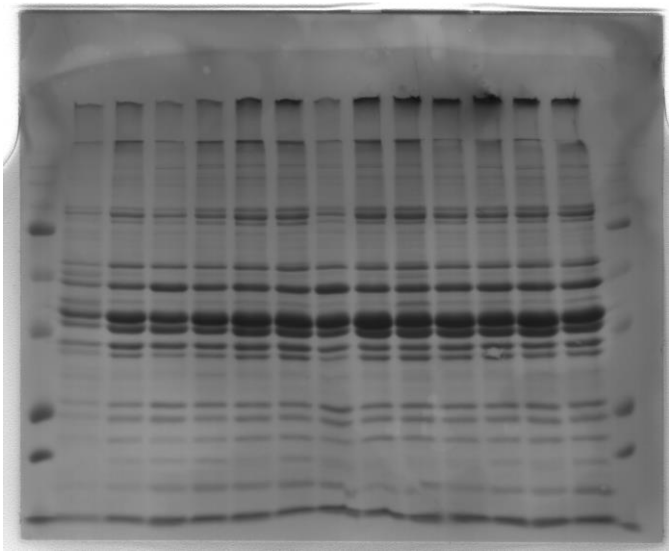

MEM 3 TOTAL PROTEIN

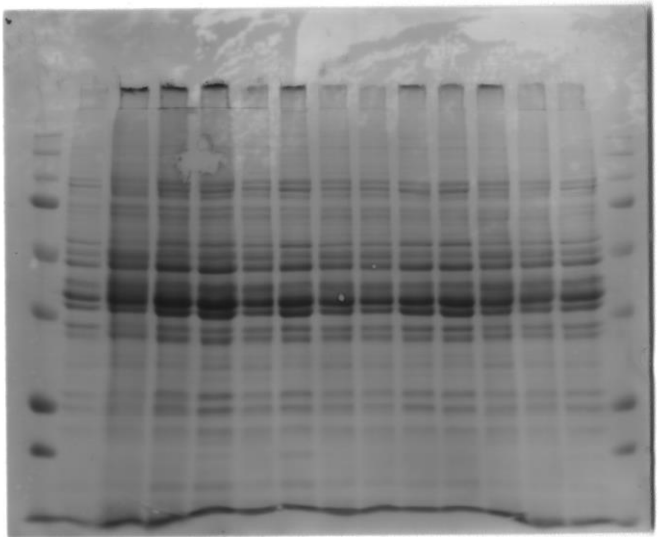

MEM 2 TOTAL PROTEIN

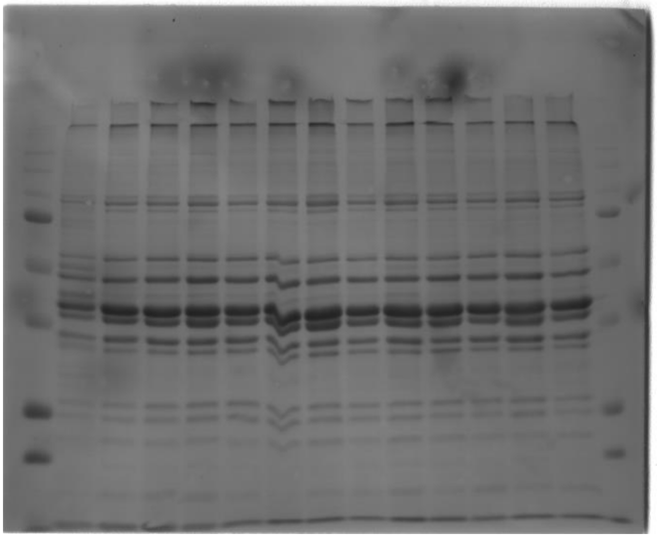

MEM 4 TOTAL PROTEIN

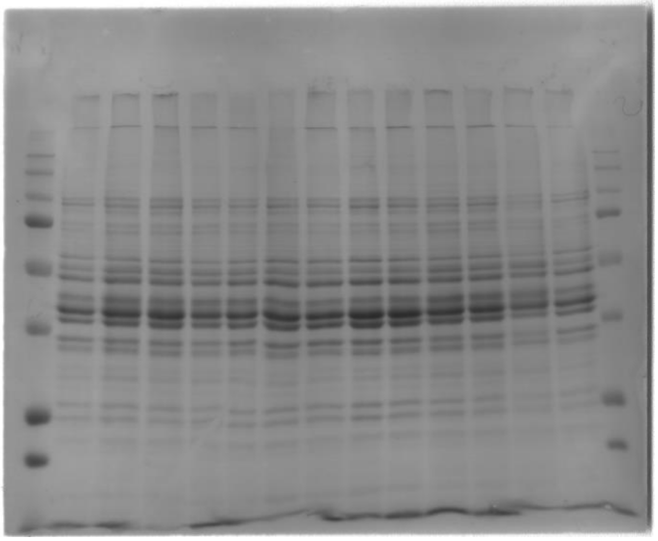

MIT 1/2

WM

MEMB 1

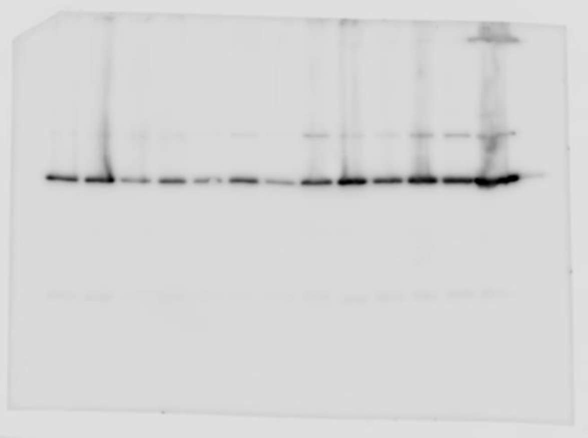

MEMB 2

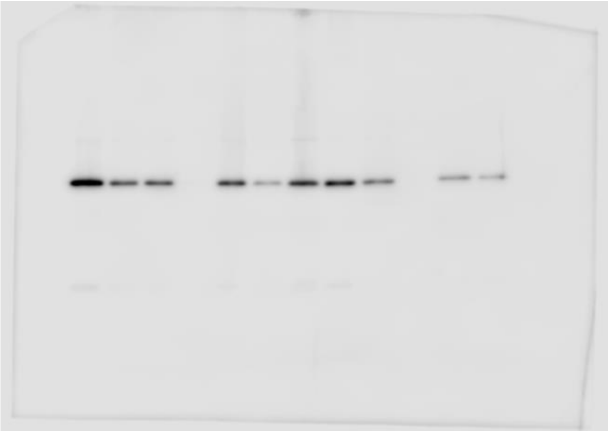

MEMB 1 TOTAL PROTEIN

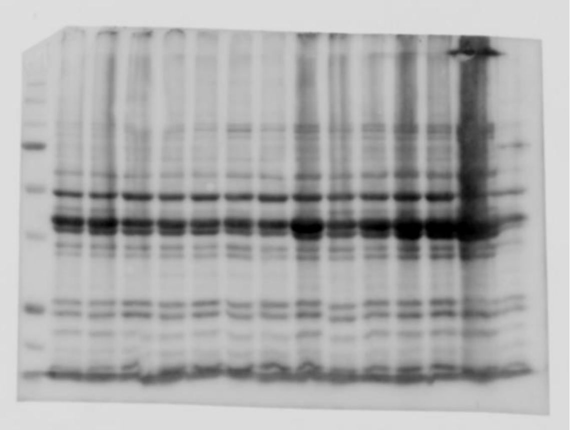

MEMB 2 TOTAL PROTEIN

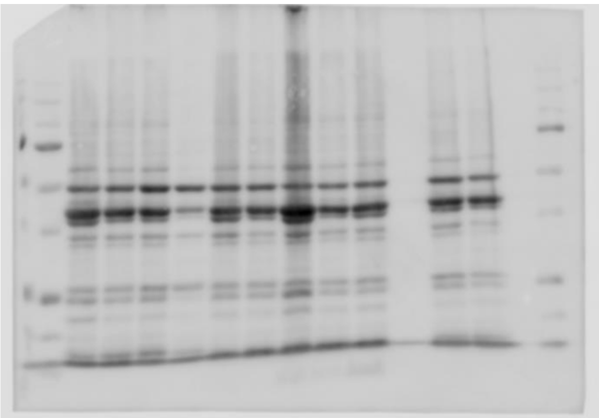

MIT 1/2

RM

MEMB 1

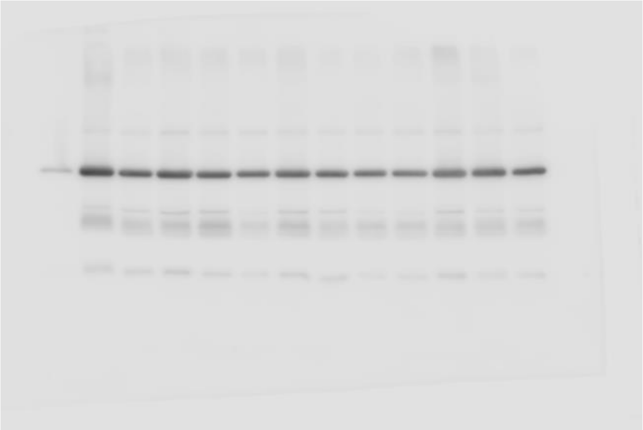

MEMB 2

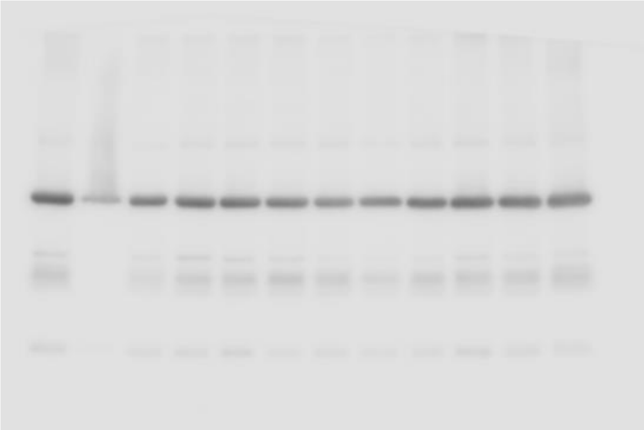

MEMB 1 TOTAL PROTEIN

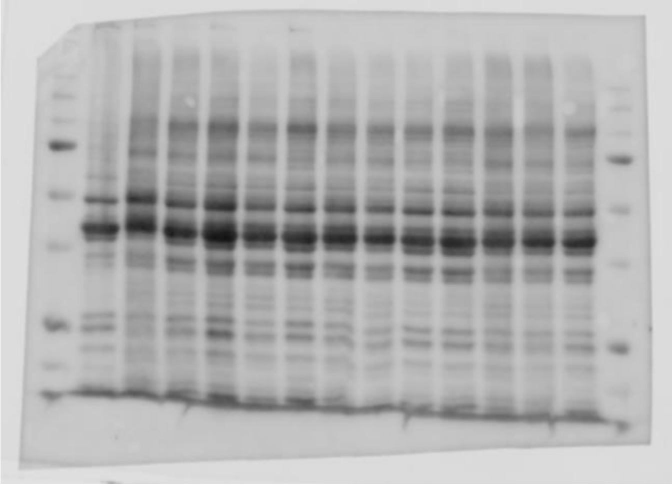

MEMB 2 TOTAL PROTEIN

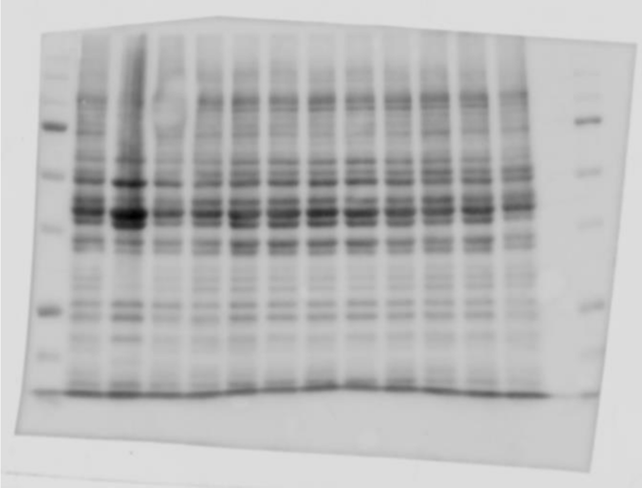

UCP3

WM

MEMB 1

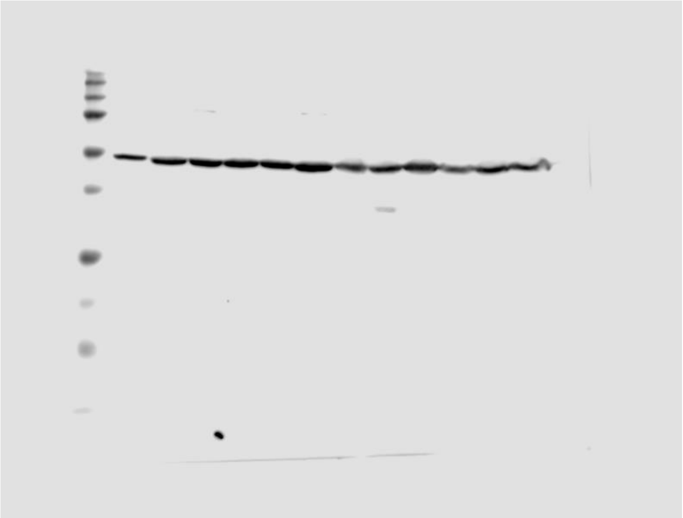

MEMB 2

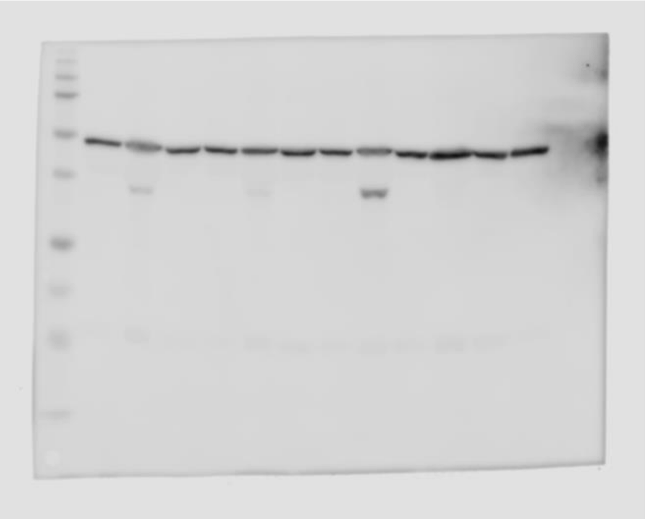

MEM 1 TOTAL PROTEIN

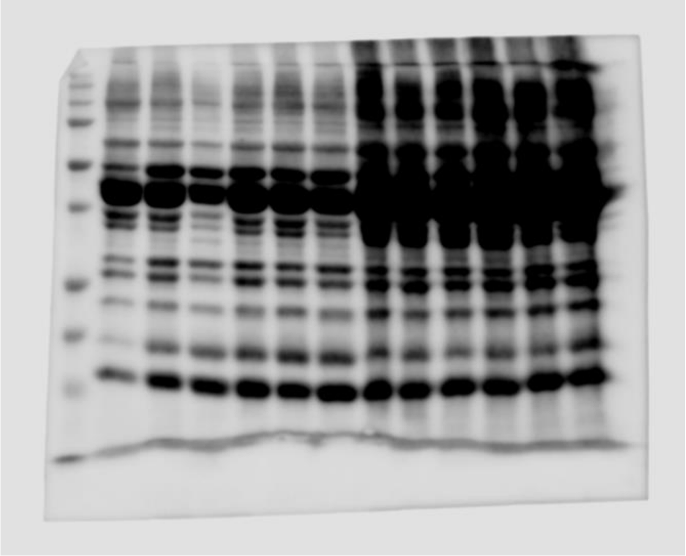

MEM 2 TOTAL PROTEIN

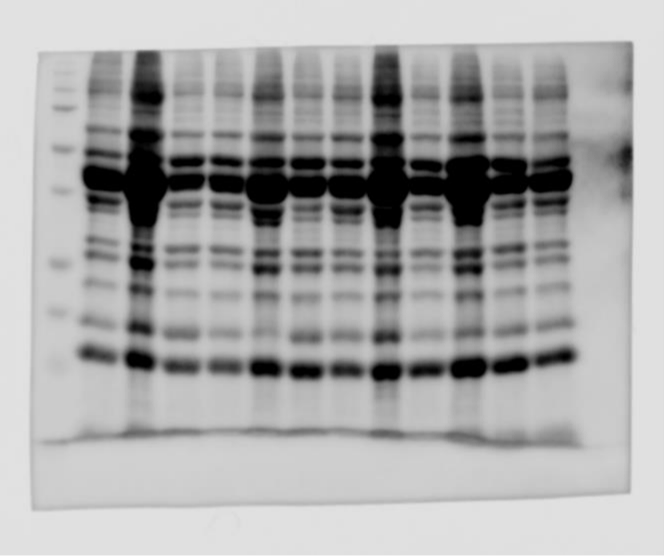

UCP3

RM

MEMB 1

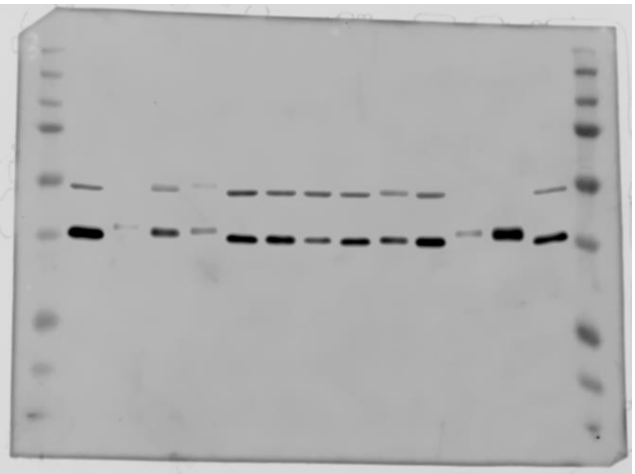

MEMB 2

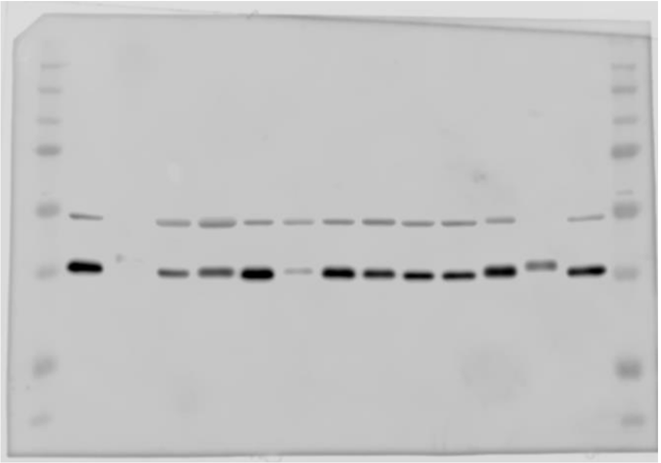

MEM 1 TOTAL PROTEIN

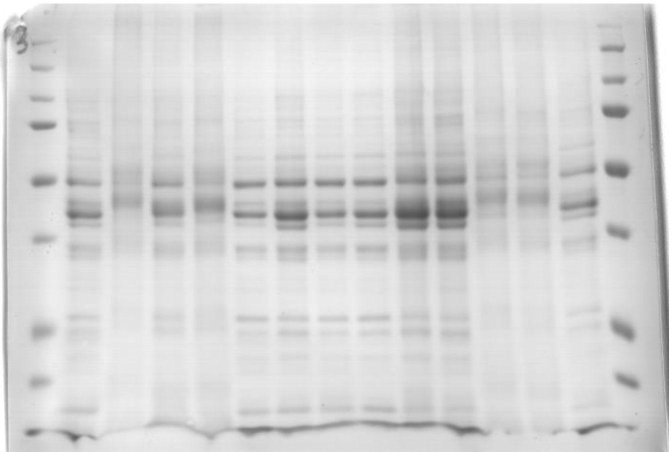

MEM 2 TOTAL PROTEIN

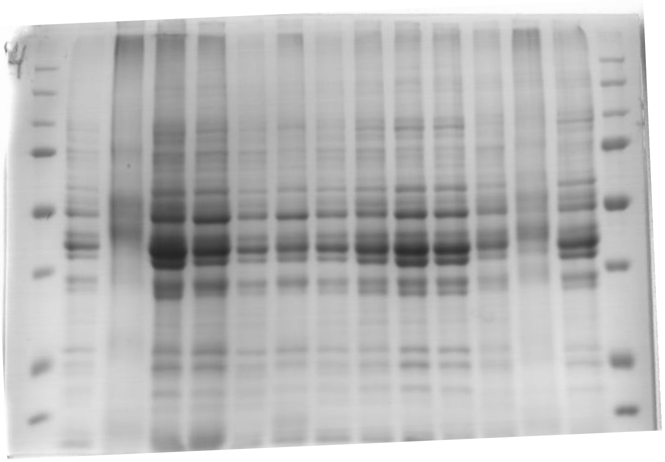

Supplement: Supplementary file 1 [file Data_Sheet_1.PDF]
